# Abstract analysis method facilitates filtering low-methodological quality and high-bias risk systematic reviews on psoriasis interventions

**DOI:** 10.1186/s12874-017-0460-z

**Published:** 2017-12-29

**Authors:** Francisco Gómez-García, Juan Ruano, Macarena Aguilar-Luque, Patricia Alcalde-Mellado, Jesús Gay-Mimbrera, José Luis Hernández-Romero, Juan Luis Sanz-Cabanillas, Beatriz Maestre-López, Marcelino González-Padilla, Pedro J. Carmona-Fernández, Antonio Vélez García-Nieto, Beatriz Isla-Tejera

**Affiliations:** 1 Department of Dermatology, Reina Sofía University Hospital, Menendez Pidal Ave, Córdoba, 14005 Spain; 2 IMIBIC/Reina Sofía University Hospital/UNiversity of Córdoba, Menendez Pidal Ave, Córdoba, 14005 Spain; 3 School of Medicine, University of Córdoba, Menendez Pidal Ave, Córdoba, 14005 Spain; 4 Department of Pharmacy, Reina Sofía University Hospital, Menendez Pidal Ave, Córdoba, 14005 Spain

**Keywords:** Systematic review, Methodological quality, Quality of reporting, AMSTAR, PRISMA for abstracts, Abstract readability, Psoriasis, Decision trees

## Abstract

**Background:**

Article summaries’ information and structure may influence researchers/clinicians’ decisions to conduct deeper full-text analyses. Specifically, abstracts of systematic reviews (SRs) and meta-analyses (MA) should provide structured summaries for quick assessment. This study explored a method for determining the methodological quality and bias risk of full-text reviews using abstract information alone.

**Methods:**

Systematic literature searches for SRs and/or MA about psoriasis were undertaken on MEDLINE, EMBASE, and Cochrane database. For each review, quality, abstract-reporting completeness, full-text methodological quality, and bias risk were evaluated using Preferred Reporting Items for Systematic Reviews and Meta-analyses for abstracts (PRISMA-A), Assessing the Methodological Quality of Systematic Reviews (AMSTAR), and ROBIS tools, respectively. Article-, author-, and journal-derived metadata were systematically extracted from eligible studies using a piloted template, and explanatory variables concerning abstract-reporting quality were assessed using univariate and multivariate-regression models. Two classification models concerning SRs’ methodological quality and bias risk were developed based on *per-item* and total PRISMA-A scores and decision-tree algorithms. This work was supported, in part, by project ICI1400136 (JR). No funding was received from any pharmaceutical company.

**Results:**

This study analysed 139 SRs on psoriasis interventions. On average, they featured 56.7% of PRISMA-A items. The mean total PRISMA-A score was significantly higher for high-methodological-quality SRs than for moderate- and low-methodological-quality reviews. SRs with low-bias risk showed higher total PRISMA-A values than reviews with high-bias risk. In the final model, only ’authors per review > 6’ (OR: 1.098; 95%CI: 1.012-1.194), ’academic source of funding’ (OR: 3.630; 95%CI: 1.788-7.542), and ’PRISMA-endorsed journal’ (OR: 4.370; 95%CI: 1.785-10.98) predicted PRISMA-A variability. Reviews with a total PRISMA-A score < 6, lacking identification as SR or MA in the title, and lacking explanation concerning bias risk assessment methods were classified as low-methodological quality. Abstracts with a total PRISMA-A score ≥ 9, including main outcomes results and explanation bias risk assessment method were classified as having low-bias risk.

**Conclusions:**

The methodological quality and bias risk of SRs may be determined by abstract’s quality and completeness analyses. Our proposal aimed to facilitate synthesis of evidence evaluation by clinical professionals lacking methodological skills. External validation is necessary.

**Electronic supplementary material:**

The online version of this article (doi:10.1186/s12874-017-0460-z) contains supplementary material, which is available to authorized users.

## Background

Therapeutic decision-making processes should be based on the best available evidence [1]. Documents that synthesise evidence concerning a particular subject facilitate access to such information for the consumers of the product in question (physicians, pharmacists, hospital committees, regulatory organisations). Systematic reviews (SRs) are the standard documents that provide syntheses of evidence. Their conclusions are often used as a starting point for the development of clinical practice guidelines, and also for establishing recommendations concerning diagnostic, prognostic, and/or therapeutic interventions [2]. However, applying the information contained within these documents requires authors to follow rigorous procedures to ensure adequate methodological quality is present, minimise the risk of bias, and facilitate reporting and dissemination. A large number of primary studies and evidence-synthesis documents have been published to date, but many are redundant, do not reach the necessary methodological quality, or have a high risk of bias [3]. Considering this situation, it is not easy for consumers to identify synthesis documents that are of good quality and have a low risk of bias.

Psoriasis is a chronic disease, with moderate and severe forms associated with significant comorbidity, impaired quality of life, and high direct and indirect costs [4]. An increasing number of elective therapies have been developed during the last decade, but these usually have potentially significant adverse side effects and high costs, which puts patients at risk and brings the sustainability of the health systems into question [5, 6]. Assessing full-text documents using Assessing the Methodological Quality of Systematic Reviews (AMSTAR) and Risk of Bias in Systematic Reviews (ROBIS) tools, we recently observed that most SRs relating to interventions in psoriasis are of low methodological quality (28.8%) and have a high bias risk (86%) [7]. However, it is impractical to suggest that interested parties apply this same method to assess the methodological quality and the risk of bias of SRs, as it is a time-consuming process that requires systematic literature searching, abstract screening, and full, in-depth manuscript assessment; further, two or more evaluators are required to control for rating discrepancies [8]. In the recent years, efforts have been made to automate some steps towards SR development. In this sense, machine learning resources have been evaluated to assist the conduction of SRs [9] as well as for assessing the risk of bias of SRs [10].

In 2013, the Preferred Reporting Items for Systematic Reviews and Meta-analyses for Abstracts (PRISMA-A) was published, featuring guidelines concerning methods of writing and presenting abstracts for systematic reviews and meta-analyses [10]. PRISMA-A is a checklist developed to help authors report all types of SRs, although it mainly relates to SRs concerning evaluations of interventions in which one or more meta-analyses are conducted. This tool features 12 items related to information that should be provided in order to present the methods, results, and conclusions in a manner that accurately reflects the core components of the full review. However, the relationship between the reporting quality of such abstracts, the methodological quality of the full texts, and the risk of bias in these texts is still unknown.

Thus, the primary objective of our study is to apply PRISMA-A to evaluate the reporting quality of SR abstracts relating to psoriasis interventions. Our secondary objective is to determine if this instrument indirectly captures the methodological quality of and the risk of bias in the full reviews, which we measured using AMSTAR and ROBIS instruments. Finally, we discuss our attempt to develop classification algorithms using PRISMA-A that can provide deeper analysis of reviews based only on abstract data.

## Methods

### Protocol and elegibility criteria

To begin, we established an a priori protocol to evaluate AMSTAR vs ROBIS in which we predict the measurement of compliance with PRISMA-A and published it in the PROSPERO International Prospective Register of Systematic Reviews (PROSPERO 2016: CRD42016053181). In this protocol, we included SRs or MAs published in scientific journals that related to interventions in skin psoriasis. Historical articles, abstracts of congresses, case reports, surveys, narrative reviews, narrative reports (i.e., reports that have a particular focus on understanding a concept), clinical practice guidelines, consensus documents, MAs performed without a systematic literature search, and reviews titled as literature reviews or integrative reviews were not included. Further, as a result of the time limitation on completing the project, the documents retrieved were restricted to English-language reviews. There was no limitation on the year of publication or study population.

### Search and selection methods

As a systematic literature search was conducted in a previous study and, taking the results listed, we filtered them to include only those published by July 5th 2016 [7]. Then, new SRs and MAs published by January 2017 were identified using MEDLINE, EMBASE, and the Cochrane Database. Details regarding the search methods applied for identifying and selecting these documents are provided in Additional file 1.

### Quality assessment of abstract reporting

Two investigators (JL-HR and JL-SC) independently assessed the abstract-reporting quality of each review; they used the same data abstraction forms for each review and were blinded to the names of the journals, the authors, and the authors’ affiliations. As mentioned above, we applied PRISMA-A, a checklist designed to determine if the content of an SR abstract is truthful, to assess reviews of psoriasis interventions [11]. PRISMA-A features a 12-item checklist concerning information that should be provided in SR abstracts; specifically, these are: title; objectives; the eligibility criteria of included studies; information sources, including key databases and dates of searches; methods of assessing bias risk; number and type of included studies; synthesis of results for main outcomes; description and direction of the effect; summary of the strengths and limitations of the evidence; general interpretation of results; funding sources, and registration number.

### Methodological quality of SRs

Two investigators (FG-G and JG-M) independently assessed the methodological quality of each review using AMSTAR tool; again, these investigators were blinded to the names of the journals, names of the authors, and authors’ criteria. In the case of a disagreement, an independent researcher (JR) was consulted. Review quality was classified by total AMSTAR score following one of the most used cutoff points for AMSTAR levels [for low (0-4), moderate (5-8), and high methodological quality (9-11) respectively [12]. Detailed information about the AMSTAR checklist and the system of rating the articles are presented in Additional file 2.

### Bias risk of SRs

Two investigators (FG-G and MA-L) independently assessed the bias risk of each review using the same data abstraction forms for each and while being blinded to the names of the journals, the names of the authors, and the authors’ affiliations; specifically, we used ROBIS, which features a four-stage approach, to assess this bias risk [11]. ROBIS is conducted over three phases. Phase 1 involves assessing the relevance of the review, and is considered optional. Phase 2 includes four domains: 1) study eligibility criteria, 2) identification and selection of studies, 3) data collection and study appraisal, and 4) synthesis and findings. Finally, phase 3 assesses the overall risk of bias in the interpretation of the review findings and whether limitations identified in any of the phase domains have been considered. To simplify analyses, SR that were rated to have an unclear risk of bias using ROBIS tool were discussed with a third evaluator to take the final decision to categorize them in the group of high or low risk bias. Recently, good validity, reliability and applicability of ROBIS tool have been demonstrated [13]. Detailed information about the ROBIS tool and the system of rating are presented in Additional file 3.

### Data extraction and statistical analysis

For studies that fulfilled the inclusion criteria, five investigators (FG-G, JG-M, PA-M, JLS-C, and MG-P) independently obtained metadata from each. Studies were then classified as Cochrane or non-Cochrane reviews. Cochrane affiliation was defined for authors of Cochrane Reviews published at the Cochrane Database of Systematic Reviews (CDSR) and authors using a Cochrane group name even if the paper was not published at CDSR. PRISMA-A results are represented on Likert scales as percentages of achievement per item. PRISMA-A results are also summarised on Likert scales in regard to methodological quality and risk of bias. Total and by item interrater reliability (IRR) of PRISMA-A was assessed using the *irr* R package. Differences in the mean total of PRISMA-A scores when comparing methodological quality and risk of bias levels were assessed using the Kruskal-Wallis and Wilkoxon tests, respectively. Evidence against the null hypothesis was considered for a two-tailed *p* value of < 0.05. Further, generalised linear models were obtained using the median total PRISMA-A score as the dependent variable. Adjustments were made for several metadata: actual observed ‘abstract word count’ (≤ 300 versus >300), ‘abstract format’ (8-headings, IMRAD, and free format), ‘Cochrane affiliation authors’, ‘number of authors’ (≤ 6 versus >6), ‘number of authors with conflict of interest’, ‘source of funding’ (pharma, academic or none/UNK), ‘PRISMA endorser journal’ (‘yes’ versus ‘no’), ‘PRISMA-A statement’ (review published before or after 2013), and ‘journal impact factor’. The ‘IMRAD’ format include: introduction, methods, results, and discussion. The ’8-headings abstract’ format includes: background, objectives, search methods, selection criteria, data collection, analysis, main results, and author’s conclusions. We checked the list of journals endorsing PRISMA at the PRISMA web (URL: http://www.prisma-statement.org/Endorsement/PRISMAEndorsers.aspx). Multivariate predictive model was created including those variables that were statistically significant in the univariate predictive models (*p*<0.05). Recursive partitioning of our dataset helped us to develop easily visualised decision rules for predicting the methodological quality of SRs based on abstract analysis. Next, two classification trees were created for methodological quality (‘high’ and ‘moderate’ levels were recoded as ‘high-moderate’ in order to produce a simpler model with a binary response) and risk of bias. Decision trees were obtained using the *rpart* R package that implements several algorithms. Cut off points were obtained as results of complex internal processes of these algorithms, and therefore they were not selected by the authors. We used cross-validation method to evaluate predictive accuracy of our model as compared with the rest of tree models. We have performed sensitivity analysis for both AMSTAR and ROBIS classification trees by random selection of the training dataset to build 2.000 models in each case. Values of ’variable importance’ parameter obtained for every node and model were plotted. Graphs were produced and statistics were analysed using several packages of R language (R Development Core Team).

### Protocol vs. overview

Our planned search strategy was recorded in PROSPERO and was compared with the final reported review methods. We decided to use the machine learning classification procedure to obtain classification trees based on PRISMA-A after our protocol was published.

## Results

### Review selection

**Fig. 1:**
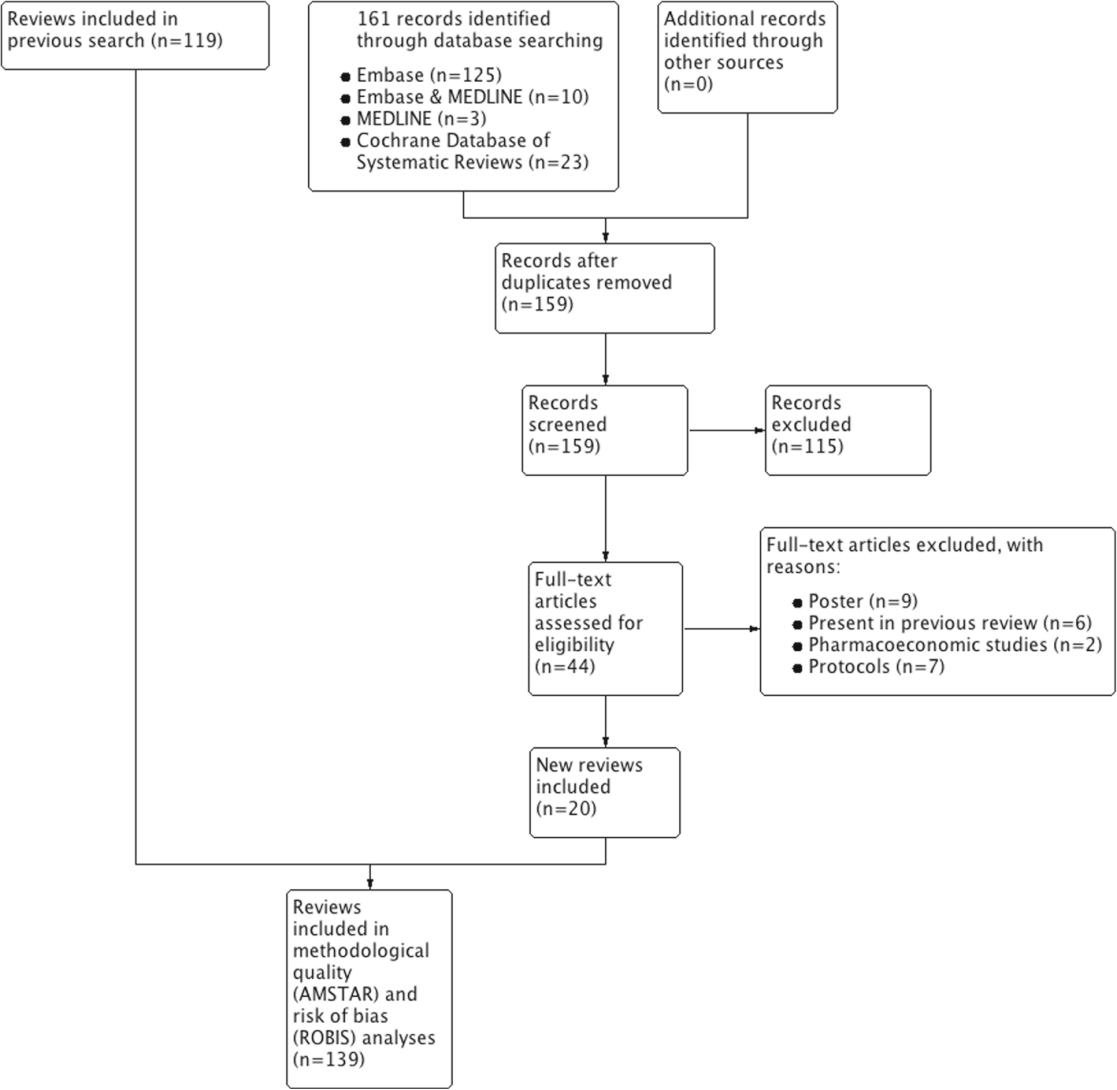
PRISMA flow diagram of article selection process

Our new database search (from July 5th 2016 to January 1st 2017) yielded 161 titles with potential relevance (125 from EMBASE & MEDLINE, 10 from EMBASE only, three from MEDLINE only, and 23 from the Cochrane Database). After excluding duplicated articles and screening titles and abstracts, 44 new studies were judged to be potentially eligible for full-text review, and after assessment, final reviews were added to the previously obtained 119 reviews (Fig. 1). Thus, 139 reviews comprising 4357 primary studies about interventions in psoriasis were published by 62 journals from 1997 to 2017. Lists of included and excluded articles are shown in Additional files 4 and 5.

### Reporting characteristics of SRs

**Fig. 2:**
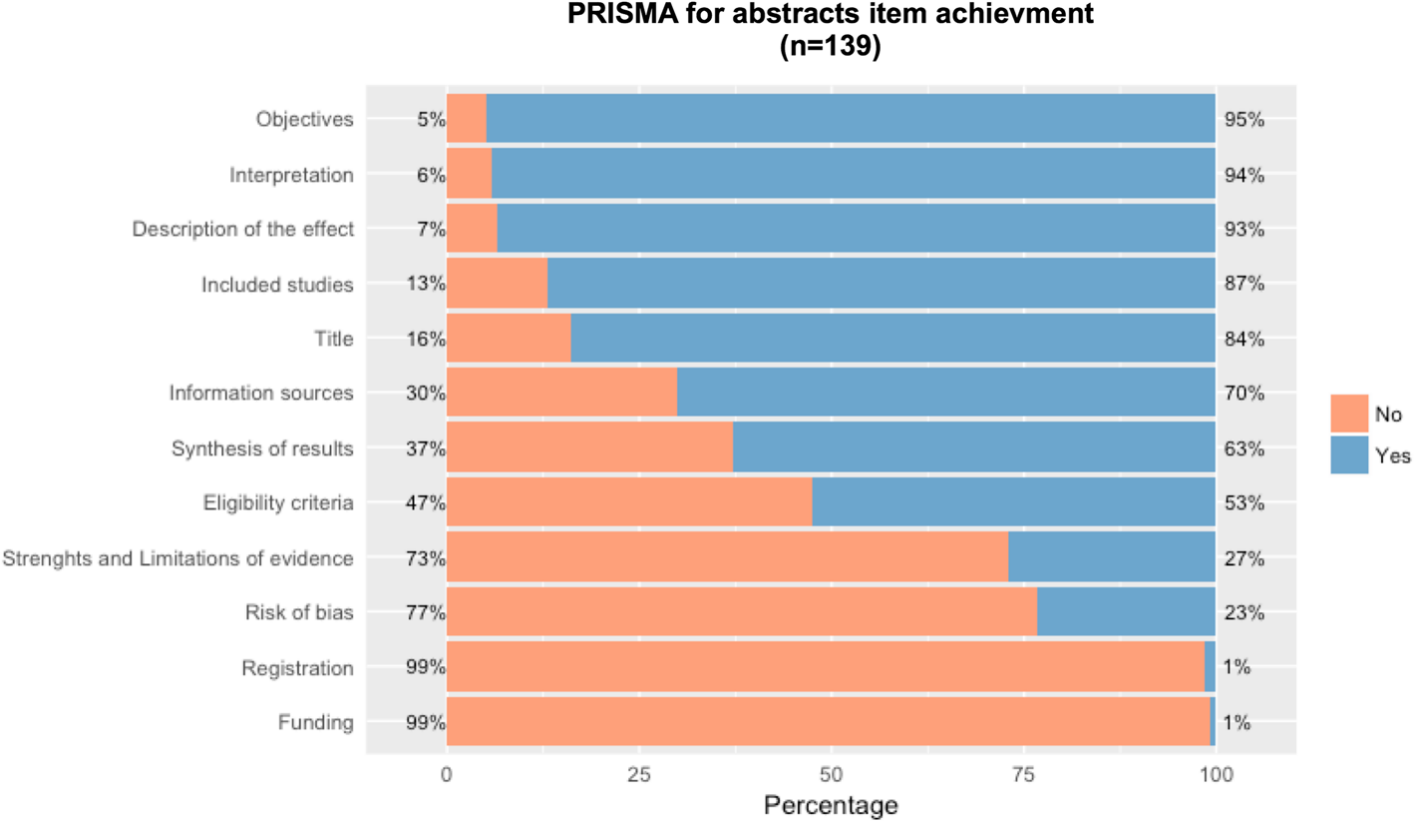
Plot of Likert scales with PRISMA-A. This graph shows the frequency distributions of responses to SR reporting assessment using *PRISMA for Abstracts*. This graph shows frequency distributions of responses (yes, no) to the 12 items of *PRISMA for Abstracts*

The interrater reliability (IRR) of both raters for total score was substantial (*κ*=0.77; 95% CI, 0.59-0.88). IRR was highest for question PEA1 (*κ*=0.86) and lowest for question PEA8 (*κ*=0.08) (Additional file 6). As shown in Fig. 2, of the 12 PRISMA-A items, there were three items for which more than 90% of the included reviews received a ‘yes’ rating: item 2 (objectives; 94.9%), item 10 (interpretation of results; 94.1%), and item 1 (description of the effect; 93.4%). However, less than 50% of the SRs fulfilled the criteria for item 5 (risk of bias; 23.3%) and item 9 (strengths and limitations of evidence; 27%). Finally, almost none of the SR abstracts fulfilled item 12 (registration; 1.4%) or item 11 (funding; 0.7%). Considering item ratings for each SR, six of the 139 reviews received a ‘yes’ rating for 10 or 11 of the 12 PRISMA-A items. The median number of fulfilled items for each review was six (range: 2-11).

### Reporting quality and risk of bias

**Fig. 3:**
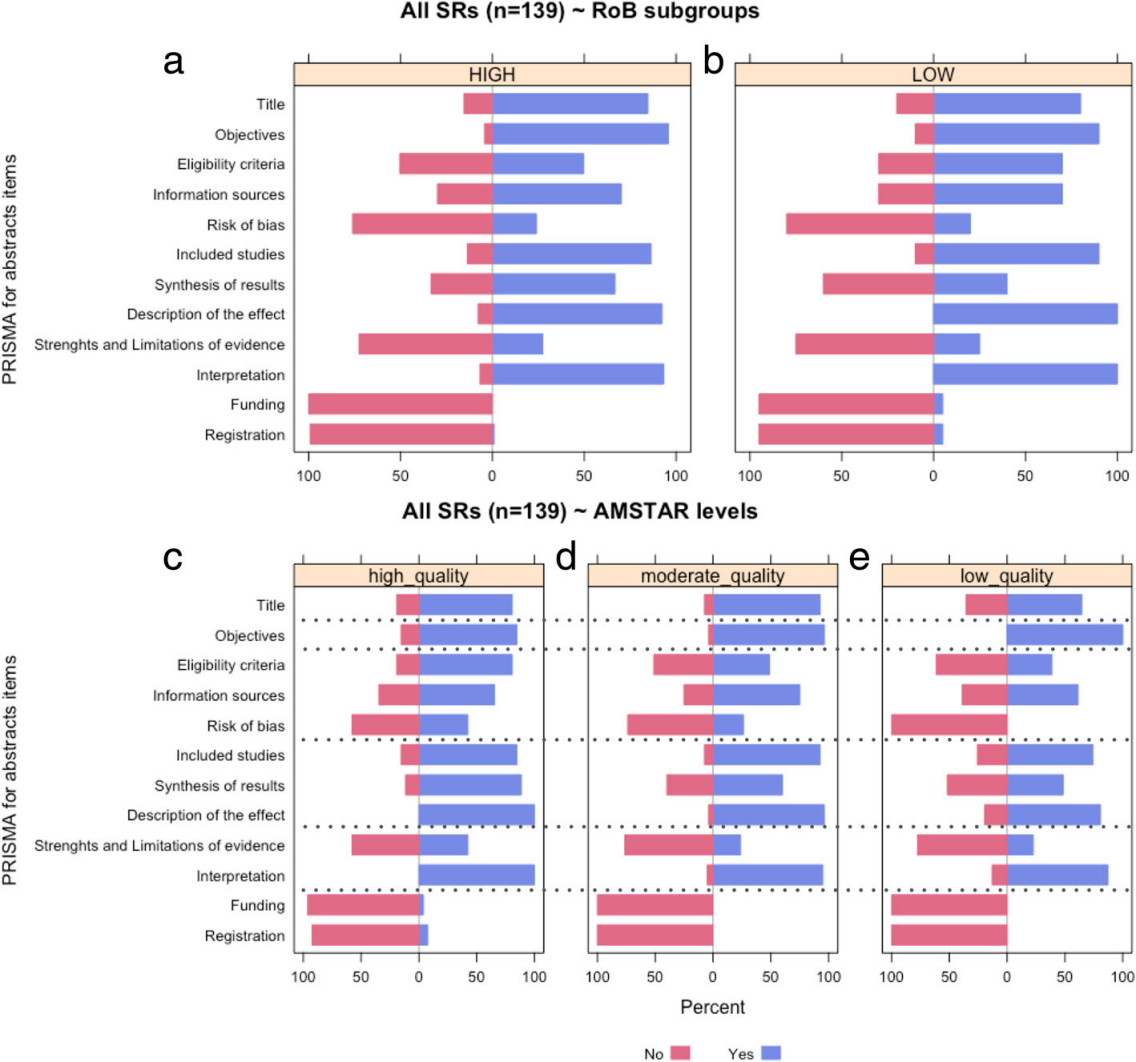
Frequency distributions of responses to reporting assessment using *PRISMA for Abstracts* comparing SR based on methodological quality and risk of bias. This panel of plots contains different graphs showing *PRISMA for Abstracts* results when reviews are subgrouped by ROBIS (**a**,**b**) and AMSTAR (**d**,**d**,**e**) classifications. (**a**-**b**) These plots display frequency distributions of responses (‘no’, ‘yes’) to *PRISMA for Abstracts* comparing reviews by risk of bias using ROBIS tool (‘high’ or ‘low’). (**c**-**d**) These plots show frequency distributions of *PRISMA for Abstracts* responses (‘no’ or ‘yes’) comparing reviews by AMSTAR-derived methodological quality levels (‘high’, ‘moderate’, or ‘low’)

For reviews with a high risk of bias, the median number of PRISMA-A items with a ‘yes’ rating was six (2-10). Interestingly, for reviews with low bias risk, the minimum number of items with a ‘yes’ rating was also six (Table 1). Fig. 3a-b shows PRISMA-A Likert scales in which the percentage of achievement per item for high-bias-risk SRs was compared with reviews that had low bias risk; this was performed using the ROBIS tool. Overall, the response profiles are quite similar, with only a slight increase of compliance found in the low-bias-risk subgroup for the ‘interpretation’, ‘funding’, and ‘registration’ items. Lastly, SRs with a low risk of bias showed higher total PRISMA-A values than reviews with high bias risk (7.7±1.26 vs 6.75±1.59, *p*=0.012) (Additional file 7).

**Table 1:** Number of PRISMA-A items reported in abstracts of SRs on psoriasis interventions classified by methodological quality (AMSTAR) or risk of bias (ROBIS)

Number of items	Methodological quality			Risk of bias	
	(AMSTAR)			(ROBIS)	
	High	Moderate	Low	High	Low
1	0	0	0	0	0
2	0	0	3	3	0
3	0	0	2	2	0
4	0	2	0	2	0
5	0	6	5	1	0
6	5	24	9	34	4
7	5	16	8	25	4
8	11	22	4	29	8
9	3	6	0	6	3
10	1	4	0	5	0
11	1	0	0	0	1
12	0	0	0	0	0

### Reporting quality and methodological quality

Figure 3c-e presents the percentage of achievement per PRISMA-A item, comparing SRs classified using the AMSTAR instrument (as high, moderate, or low methodological quality). In this case, unlike the findings concerning the bias-risk subgroups, there are different patterns for each level of methodological quality. For high-methodological-quality reviews, the median number of items with a ‘yes’ rating was eight (6-11), with six (4-10) and five (2-8) for moderate and low quality reviews, respectively (Table 1). Item 5, ‘risk of bias’, showed the widest variation between the subgroups, and items 10 (‘funding’) and 11 (‘registration’) displayed minimal variation. Lastly, the mean total PRISMA-A score was significantly higher for SRs with high methodological quality than for moderate (7.73±0.13 vs 7.05±0.13, *p*=0.031) and low methodological quality (7.73±0.13 vs 5.77±0.13, *p*=0.001) (Additional file 8).

### Factors influencing reporting quality

Univariable and multivariable logistic ordinal regressions were performed in order to predict PRISMA-A results (Table 2). The univariable regression models showed ‘abstract word count > 300’, ‘Cochrane author affiliation’, ‘authors per review > 6’, and ‘academic source of funding’ to be predictors of high achievement in regard to PRISMA-A items; meanwhile, IMRAD and free abstract formats were predicted to suggest a lower number of PRISMA-A items than the 8-heading abstract format. Journals with an impact factor of ≤ 3 or journals that did not endorse PRISMA-A statements were also used as predictors for low reporting scores. In the final model, only ‘authors per review > 6’ (OR: 1.098; 95% CI: 1.012-1.194), ‘academic source of funding’ (OR: 3.630; 95% CI: 1.788-7.542), and ‘PRISMA-endorsed journal’ (OR: 4.370; 95% CI: 1.785-10.98) predicted PRISMA-A variability.

**Table 2:** Univariate and multivariate predictive models of PRISMA-A items reported in abstracts of SRs on psoriasis interventions

		Univariable			Multivariable
Variables		analysis			analysis
	n	Estimate (SE)	*p*-value	Estimate (SE)	OR (95%CI)
Abstract word count					
≤ 300	98	1			
> 300	40	1.068(0.539)	0.049	0.741(0.332)	1.456(0.658-3.240)
Abstract format					
8-headings	16	1			
IMRAD	77	− 1.205(0.418)	0.004	− 0.049(0.418)	0.951(0.420-2.153)
Free format	45	− 1.539(0.445)	< 0.001	− 0.152(0.415)	0.858(0.378-1.939)
Cochrane affiliation					
Yes	9	1.068(0.539)	0.049	0.543(0.734)	1.722(0.401-7.296)
No	129	1			
Number of authors					
≤ 6	102	1			
> 6	36	0.600(0.303)	0.049	0.725(0.341)	1.098(1.012-1.194)
Conflict of interest					
≤ 20*%*	59	1			
> 20%	61	− 0.400(0.260)	0.126		
Funding source					
Academic	39	1.156(0.283)	< 0.001	1.247(0.428)	3.630(1.788-7.542)
Pharmaceutical	40	− 0.198(0.297)	0.506		
No funding/UNK	59	0.302(0.478)	0.528		
PRISMA endorser journal					
Yes	10	1.562(0.448)	0.002	2.016(0.698)	4.370(1.785-10.98)
No	117	1			
PRISMA-A statement					
Review published before	71	− 0.421(0.371)	0.119		
Review published after	68	1			
Journal impact factor					
≤ 3	74	1			
> 3	43	0.331(0.373)	0.014	0.331(0.373)	1.392(0.669-2.912)

### Classification trees for SRs methodological quality prediction based on abstract reporting assessment

**Fig. 4:**
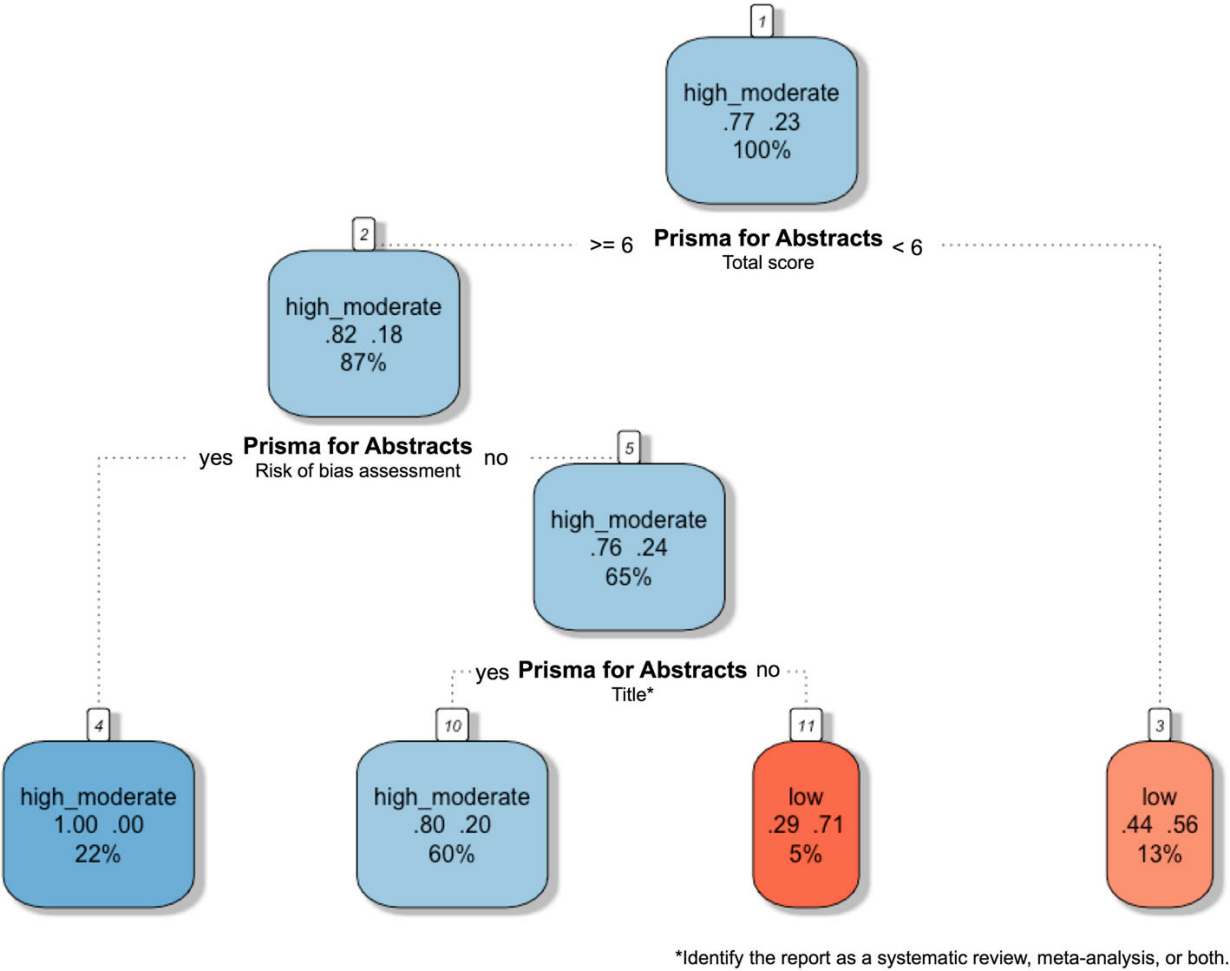
Tree classification model of the methodological quality of SRs based on PRISMA-A total and per item scores. Each node shows from top to bottom the predicted class (high-moderate, low), the predicted probability of each class, and the percentage of observations in the node

**Fig. 5:**
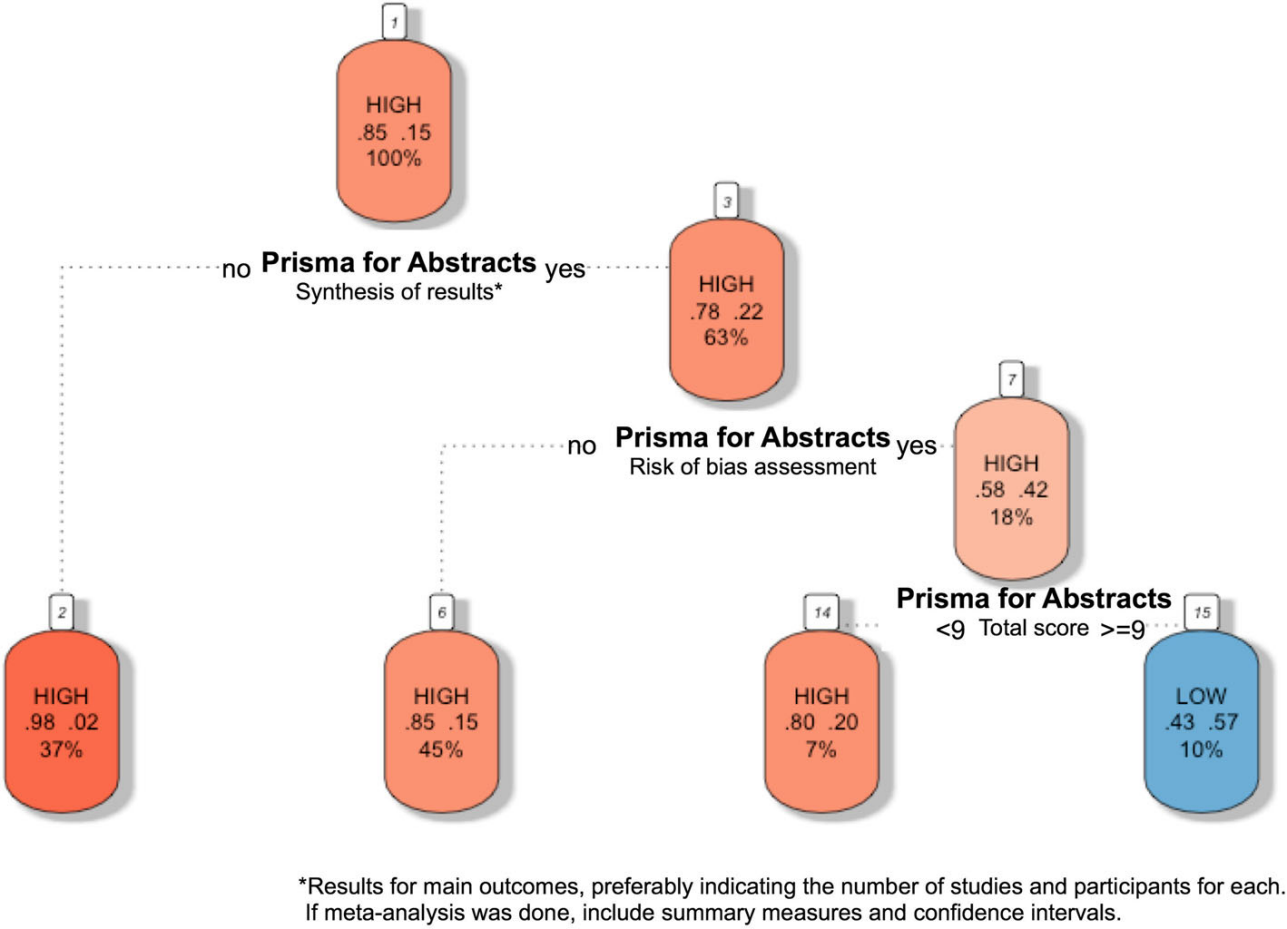
Tree classification model of the bias risk of SRs based on PRISMA-A total and per item scores. Each node shows from top to bottom the predicted class (high, low), the predicted probability of each class, and the percentage of observations in the node

We used classification trees as a visual tool with which to gain an idea of the abstract-related variables that are important for predicting SRs with low methodological quality and high bias risk, and how they relate to each other; this was because trees can capture nonlinear relationships among predictors. Total and by-item results for PRISMA-A were included as predictor variables. Figures 4 and 5 display pruned classification trees for both methodological quality and risk of bias, respectively. Essentially, Fig. 4 shows that abstracts that had a total PRISMA-A score of less than six, lacking any identification in the title of being an SR or MAs, as well as lacking an explanation of the methods applied for assessing bias risk, were classified using AMSTAR as having low-methodological quality with a root node error of 0.15 and a misclassification rate of 22.6% in the cross-validation. In Fig. 5, abstracts with a total PRISMA-A score equal to or higher than nine which included the results of the main outcomes and an explanation concerning the methods used for assessing bias risk were classified as having low-bias risk, with a root node error of 0.14 and a misclassification rate of 20.6% in the cross-validation. We found that the nodes included in our tree models were also at the top ranking of nodes when ordered by median importance after sensitivity analysis (Additional files 9 and 10). Overall, a higher dispersion of ‘variable importance’ values of AMSTAR-derived trees as compared with ROBIS trees suggests that AMSTAR classification tree is less robust than ROBIS classification tree.

## Discussion

### Main findings

To the best of our knowledge, this is the first study to evaluate the capacity of PRISMA-A to determine the methodological quality and bias risk of SRs or MAs relating to psoriasis interventions. In short, this study suggests that the reporting quality of abstracts of reviews published concerning psoriasis interventions is suboptimal. Overall, the average percentage of PRISMA-A items featured in each abstract was 50-67%. While ‘objectives’, ‘interpretation of results’, and ‘description of effect’ were included in almost all abstracts, the majority failed to adequately report ‘strengths and limitations’ and ‘risk of bias’; furthermore, registration numbers and disclosures of sources of funding were almost universally absent.

We found that methodological quality and risk of bias, assessed using AMSTAR and ROBIS instruments, correlated positively with the PRISMA-A evaluations of the quality and completeness of abstract reporting. Previous studies have supported the theory that improving the abstract quality of SRs may provide a more accurate reflection of their methodological quality. Previous studies, applying AMSTAR, evaluated the quality of SRs with regard to adherence to PRISMA statements, and found that PRISMA endorsement enhanced compliance with AMSTAR scale items in gastroenterology/hepatology and surgical journals [14, 15]. Further, using a masked randomised trial, Cobo et al. analysed the feasibility of using CONSORT- and STROBE-reporting guidelines to support the peer-review process performed by a general medicine journal editorial team [16]. Moreover, Rice et al., using AMSTAR, found a positive correlation between the overall quality ratings of SRs with MAs and the number of PRISMA-A items adequately reported [17].

The above findings are similar to our own, as we also found that the methodological quality of reviews assessed using the AMSTAR instrument correlated positively with PRISMA-A evaluations of the quality and completeness of abstract reporting. However, no study has yet been published presenting a significant correlation between PRISMA-A compliance and risk of bias; in our study, significant differences in terms of abstract quality were observed between SRs with high and low bias risk.

### Strengths and limitations

In this study, we explored, for the first time, the capacity of PRISMA-A to determine both the methodological quality and the bias risk of full-text reviews using ROBIS and AMSTAR tools. Our study includes a large sample of over 15 years of reviews (*n*=139) concerning interventions in psoriasis. The study was performed using a systematic search strategy and following an a priori protocol published in PROSPERO; the AMSTAR and ROBIS assessments were performed independently by two authors, and there were few disagreements during the process, all of which were solved through discussion. Nevertheless, our study has some limitations. First, this study only featured SRs and MAs relating to interventions in psoriasis, so there is a limitation in terms of the generalisability of the data, as we did not compare our results to reviews conducted in relation to other diseases or areas of healthcare. Second, the search was restricted to MEDLINE, EMBASE, and the Cochrane database; this was because our intention was to obtain a representative sample of published systematic reviews concerning psoriasis interventions, rather than cover all such reviews. We did not search for SRs in grey literature databases, and, therefore, we cannot establish differences in terms of methodological quality and risk of bias with respect to those that were examined. Third, during the cross-validation, we found a misclassification rate of 20-22%; this means that for one in five abstracts, the methodological quality and risk of bias are mistakenly classified. To rectify this, we would require external validation to test the performance of our models with other datasets. In any case, a desirable improvement in the quality of reporting could result in the disambiguation of many SRs classified as having moderate quality and causing level overlapping during the cross-validation. Fourth, a limitation of this work is that different reviewers applied PRISMA-A, AMSTAR and ROBIS. Only one of threes raters carried out the evaluations both with AMSTAR and ROBIS tools. Although their results were compared in pairs and discrepancies were discussed with a fourth rater, there is a risk that this issue will affect the validity of our results. Finally, it is a limitation not to have considered the year in which journals are endorsing PRISMA-A and there is a risk of bias in this regard.

### Our findings in context

Our findings were similar to those of a previous study conducted by Bigna et al. In this latter study, the authors found that the quality of reporting was declining in terms of the ‘strength and limitations of evidence’ and ‘funding’ of reviews [18]. Further, Tsou et al. used PRISMA-A to analyse 200 randomly selected abstracts of SRs relating to health interventions and found that less than 50% of the abstracts contained information concerning the ‘risk of bias assessment’ (23%), ‘study protocol registration’ (2%), and ‘funding source’ (1%) [19]. Moreover, Seehra et al. studied the reporting completeness of abstracts of SRs published in dental speciality journals [20]. They developed a check list that included several items from different sources: PRISMA statement guidelines [21], the Cochrane Handbook for Systematic Reviews of Interventions [22], and the paper by Beller et al. [14]. We did not find quality of reporting differences between reviews as they were published before vs after PRISMA-A statement. Our results are similar to those found by Panic et al. [23]. These authors demonstrated that the quality of reporting improved only sub-optimally in the years following the publication of PRISMA.

The capacity of abstract extension to predict PRISMA-A variability has also been addressed in other studies. Interestingly, the number of words per summary explains a very small part of it [17], and even better reporting results were observed for abstracts with < 300 words [16]. In the latter study, a better abstract structure (8-headings vs IMRAD formats) also predicted an improved reporting quality. These results are similar to ours and suggest that abstract systematization and concretion are more important than its extension to define the quality of the summary report.

### Implications of results

Motivated by the possibility of capturing through an abstract, at least in part, the methodological quality and the risk of bias of a study, which are normally evaluated using information contained in the full text of the document, we explored the possibility of obtaining simplistic and feasible decision models that are easy to interpret and intuitive to follow. Our method is offered as a support to decision making and does not intend to replace the rigorous final analysis of each synthesis document, but it allows to prioritize in a simple and rapid way those documents obtained in a first search by professionals not experts in this type of methodology. We believe that the information contained in the abstract is a good source that can allow us to work in this sense and this is the original contribution that we make. The importance of our proposed tree models lies in their capacity to assist in abstract filtering using just the predicted methodological quality and bias risk determined through PRISMA-A abstract analysis, which is a more feasible instrument than the AMSTAR or ROBIS tools. Our decision trees have been constructed using a machine learning tool. This type of technology is currently being used to systematize some aspects of RS such as article selection or risk assessment bias [9]. We believe that the association of validated tools that measure quality or bias risk and machine learning technology may improve methodological assessment processes. Better meta-epidemiological knowledge together with the development of text mining strategies will allow to develop models that help clinicians to simplify making decisions at clinical setting. Finally, the final classification determined in both decision trees is congruent with the idea that methodological quality explains only part of the risk bias of SRs, as we found the degree of compliance with PRISMA-A required to predict SRs of low risk bias is greater than that required to predict high-methodological-quality SRs. Therefore, we can conclude that the methodological quality and the risk of bias of SRs may be captured by analysing the quality and completeness of abstract reporting, and that by applying our decision tree models, the review-filtering process may be improved through rapid abstract analysis.

## Conclusions

Our proposal is aimed to facilitate the evaluation of evidence synthesis by clinical professionals with a lack of methodological knowledge and skills. It does not intend to replace the rigorous final analysis of each review, but it allows to prioritize in a simple and rapid way those documents obtained in a first search. We believe that summaries are a good source to investigate methodological quality and risk of bias through quality and completeness assessment of abstracts. We are aware that our decision trees could be improved and that a external validation of our models in different research fields is necessary.

## Additional files

Additional file 1 **Appendix 1.** Supplementary materials and methods. (DOC 23 kb)

Additional file 2 **Appendix 4.** AMSTAR checklist. (DOC 25 kb)

Additional file 3 **Appendix 5.** Phase 2 domains and signaling questions of ROBIS tool. (DOC 32 kb)

Additional file 4 **Appendix 2.** List of included studies. (DOC 86 kb)

Additional file 5 **Appendix 3.** List of non included studies and reasons for exclusion. (DOC 733 kb)

Additional file 6 **Appendix 6.** PRISMA-A IRR using Fleiss’ Kappa for two raters. (DOC 16 kb)

Additional file 7 **Supplementary graph S1.** Analysis of total PRISMA-A scores by risk of bias levels. (TIFF 17203 kb)

Additional file 8 **Supplementary graph S2.** Analysis of total PRISMA-A scores by methodological quality levels. (TIFF 17203 kb)

Additional file 9 **Supplementary graph S3.** Ranked variable importance based on mean values after running 2000 random AMSTAR-based classification tree models. (TIFF 1249 kb)

Additional file 10 **Supplementary graph S4.** Ranked variable importance based on mean values after running 2000 random ROBIS-based classification tree models. (TIFF 1249 kb)

## Abbreviations

AMSTAR: Assessing the methodological quality of systematic reviews
CI: Confidence interval
IMRAD: Introduction, methods, results, and discussion
MAs: Meta-analyses
OR: Odds ratio
PRISMA: Preferred reporting items for systematic reviews and meta-analyses
PRISMA-A: PRISMA extension for abstracts
RCTs: Randomized controlled trials
RoB: Risk of bias
SRs: Systematic reviews

## References

[1] Dias S, Welton NJ, Sutton AJ, Ades AE (2013). Evidence synthesis for decision making 1: introduction. Med Decis Making.

[2] Abuabara K, Freeman EE, Dellavalle R (2012). The role of systematic reviews and meta-analysis in dermatology. J Invest Dermatol.

[3] Ioannidis JP (2016). The Mass Production of Redundant, Misleading, and Conflicted Systematic Reviews and Meta-analyses. Milbank Q.

[4] Goff KL, Karimkhani C, Boyers LN, Weinstock MA, Lott JP, Hay RJ, Coffeng LE, Norton SA, Naldi L, Dunnick C, Armstrong AW, Dellavalle RP (2015). The Global Burden of Psoriatic Skin Disease. Br J Dermatol.

[5] Gómez-García F, Epstein D, Isla-Tejera B, Lorente A, Vélez García-Nieto A, Ruano J (2017). Short-term efficacy and safety of new biological agents targeting the interleukin-23-T helper 17 pathway for moderate-to-severe plaque psoriasis: a systematic review and network meta-analysis. Br J Dermatol.

[6] Nast A, Jacobs A, Rosumeck S, Werner RN (2015). Efficacy and Safety of Systemic Long-Term Treatments for Moderate-to-Severe Psoriasis: A Systematic Review and Meta-Analysis. J Invest Dermatol.

[7] Gomez-Garcia F, Ruano J, Gay-Mimbrera J, Aguilar-Luque M, Sanz-Cabanillas JL, Alcalde-Mellado P, Maestre-Lopez B, Carmona-Fernandez PJ, Gonzalez-Padilla M, Velez Garcia-Nieto A, Isla-Tejera B (2017). Most systematic reviews of high methodological quality on psoriasis interventions are classified as high risk of bias using ROBIS tool. J Clin Epidemiol.

[8] Borah R, Brown AW, Capers PL, Kaiser KA (2017). Analysis of the time and workers needed to conduct systematic reviews of medical interventions using data from the PROSPERO registry. BMJ Open.

[9] Hempel S, Shetty KD, Shekelle PG, Rubenstein LV, Danz MS, Johnsen B, Dalal SR (2012). Machine Learning Methods in Systematic Reviews: Identifying Quality Improvement Intervention Evaluations [Internet].

[10] Millard LA, Flach PA, Higgins JP (2016). Machine learning to assist risk-of-bias assessments in systematic reviews. Int J Epidemiol.

[11] Whiting P, Savovic J, Higgins JP, Caldwell DM, Reeves BC, Shea B, Davies P, Kleijnen J, Churchill R, ROBIS group (2016). ROBIS: A new tool to assess risk of bias in systematic reviews was developed. J Clin Epidemiol.

[12] Interventions Directed to Consumers. [cited 9 October 2017];Canadian Agency for Drugs and Technologies in Health (CADTH) 2014. Available from: https://www.cadth.ca/interventions-directed-professionals.

[13] Bühn S, Mathes T, Prengel P, Wegewitz U, Ostermann T, Robens S, Pieper D (2017). The risk of bias in systematic reviews tool showed fair reliability and good construct validity. J Clin Epidemiol.

[14] Beller EM, Glasziou PP, Altman DG (2013). PRISMA for Abstracts: Reporting Systematic Reviews in Journal and Conference Abstracts. PLoS Med.

[15] Chapman SJ, Drake TM, Bolton WS, Bernard J, Bhangu A (2017). Longitudinal analysis of reporting and quality of systematic reviews in high-impact surgical journals. Br J Surg.

[16] Cobo E, Cortés J, Ribera JM, Cardellach F, Selva-O’Callaghan A (2011). Effect of using reporting guidelines during peer review on quality of final manuscripts submitted to a biomedical journal: masked randomised trial. BMJ.

[17] Rice DB, Kloda LA, Shrier I, Thombs BD (2016). Reporting quality in abstracts of meta-analyses of depression screening tool accuracy: a review of systematic reviews and meta-analyses. BMJ Open.

[18] Bigna JJ, Um LN, Nansseu JR (2016). A comparison of quality of abstracts of systematic reviews including meta-analysis of randomized controlled trials in high-impact general medicine journals before and after the publication of PRISMA extension for abstracts: a systematic review and meta-analysis. Syst Rev.

[19] Tsou AY, Treadwell JR (2016). Quality and clarity in systematic review abstracts: an empirical study. Res Synth Methods.

[20] Seehra J, Fleming PS, Polychronopoulou A, Pandis N (2013). Reporting completeness of abstracts of systematic reviews published in leading dental specialty journals. Eur J Oral Sci.

[21] Liberati A, Altman DG, Tetzlaff J, Mulrow C, Gøtzsche PC, Ioannidis JPA (2009). The PRISMA statement for reporting systematic reviews and meta-analyses of studies that evaluate healthcare interventions: explanation and elaboration. BMJ.

[22] Higgings JPT, Green S, (eds).Cochrane handbook for systematic reviews of interventions 5.1.0 [updated March 2011], The Cochrane Colaboration, 2011. Available at: http://handbook-5-1.cochrane.org/. Accessed 5 May 2017.

[23] Panic N, Leoncini E, de Belvis G, Ricciardi W, Boccia S (2013). Evaluation of the endorsement of the preferred reporting items for systematic reviews and meta-analysis (PRISMA) statement on the quality of published systematic review and meta-analyses. PLoS One.

