# Supplementary material for: Abstract analysis method facilitates filtering low-methodological quality and high-bias risk systematic reviews on psoriasis interventions
Source: BMC Med Res Methodol. 2017 Dec 29;17:180. doi: 10.1186/s12874-017-0460-z (PMC5747101; doi:10.1186/s12874-017-0460-z)
Supplement: Supplementary file 1 — Appendix 1. Supplementary materials and methods. (DOC 23 kb) [file 12874_2017_460_MOESM1_ESM.doc]

**Title**: Abstract analysis method facilitates filtering low-methodological quality and high-bias risk systematic reviews on psoriasis interventions

**Authors**: Francisco Gómez-García, Juan Ruano, Macarena Aguilar-Luque, Patricia Alcalde-Delgado, Jesús Gay-Mimbrera, José Luis Hernández-Romero, Juan Luis Sanz-Cabanillas, Beatriz Maestre-López, Marcelino González-Padilla, Pedro J. Carmona-Fernández, Antonio Vélez García-Nieto, and Beatriz Isla-Tejera

**Supplementary materials and methods.**

**Search methods of reviews**

A search of SRs and MAs protocol registries including PROSPERO and the Cochrane Database of Systematic Reviews revealed that no similar studies were underway as of May 2016. SRs and MAs published up to 4th July 2016 were identified in MEDLINE, EMBASE, and the Cochrane Database by a comprehensive systematic Boolean search with MeSH terms 'psoriasis'/exp or psoriasis) and 'meta analysis' or 'systematic review' . We identified additional eligible studies by searching the reference lists of included SRs, MAs, and health technology assessment (HTA) reports. We contacted study authors when necessary to identify further information that we may have missed.

**Methods for identification and selection of reviews**

Some authors independently performed all tasks for study filtering and selection (FG-G, MAL, BML, and PJGF) and data extraction (FG-G, JG-M, JLSC, and MG-P). The screening was performed in two stages. In the first stage, abstracts downloaded from the literature searches were screened by two reviewers. Reports of systematic reviews were considered eligible for inclusion if the terms or phrases “systematic review”, “meta-analysis” or “overview” were used in the title or abstract, or if the main text provided a clear indication that a systematic review had been carried out. Any study clearly not meeting the eligibility criteria was rejected. In the second stage, full papers were retrieved for the selected candidate study and assessed by two reviewers to identify all SRs and MAs meeting the eligibility criteria. Since AMSTAR evaluates the quality of the SRs and one of the items is about the analysis performed on them, we only select the papers, which in our opinion, either in the methodology section or in the supplementary material provided sufficient information that ensured that a systematic review was carried out. In doubtful or controversial cases, all identified discrepancies at first stage and throughout were resolved through by discussion and in select cases by involving a different investigator (JR).

###### **Assessment of methodological quality of included reviews**

The 11 criteria of the AMSTAR were rated as “yes”(criteria were met), “no” (criteria were not met), “cannot answer” (unclear information) or “not applicable” (criteria could not be evaluated because of the design of background studies in the reviews) (**eTable 4 of Supplementary Material**). For all items except item 4, ratings of “yes” were scored 1, and ratings of “no”, “cannot answer” and “not applicable” were scored 0. For item 4, a rating of ’no’ (that is, the review did not exclude unpublished or grey literature) is considered adequate. The highest possible AMSTAR score is 11. Review quality was classified by AMSTAR score following quality levels with similar cutoff points used by most of studies [for low (0-4), moderate (5-8), and high methodological quality (9-11) respectively]. Data were analyzed from August 30th to September 15th, 2016. A 10-study pilot evaluation were performed prior to evaluation of the selected articles in order to standardize use and eliminate inconsistencies. Quality assessment discrepancies were discussed with a third author (JR) until an agreement was reached.

###### Data Analysis

We used a range of approaches to present the results of included reviews. We captured article and journal metadata using standardized data extraction templates implemented in AppSheet, a custom mobile app based on Google forms(4)⁠. The agreement between the two raters was tested using the Cohens’s kappa (for squared) with the *irr* R package. Values of *kappa* can range from -1.0 to 1.0, with -1.0 indicating perfect disagreement below chance, 0.0 indicating agreement equal to chance, and 1.0 indicating perfect agreement above chance. A rule of thumb is that a *kappa* of .70 or above indicates adequate interrater agreement. A value of 0.65 or greater was chosen for sufficient agreement.
