# Supplementary material for: Abstract analysis method facilitates filtering low-methodological quality and high-bias risk systematic reviews on psoriasis interventions
Source: BMC Med Res Methodol. 2017 Dec 29;17:180. doi: 10.1186/s12874-017-0460-z (PMC5747101; doi:10.1186/s12874-017-0460-z)
Supplement: Supplementary file 2 — Appendix 4. AMSTAR checklist. (DOC 25 kb) [file 12874_2017_460_MOESM2_ESM.doc]

**Title**: Abstract analysis method facilitates filtering low-methodological quality and high-bias risk systematic reviews on psoriasis interventions

**Authors**: Francisco Gómez-García, Juan Ruano, Macarena Aguilar-Luque, Patricia Alcalde-Delgado, Jesús Gay-Mimbrera, José Luis Hernández-Romero, Juan Luis Sanz-Cabanillas, Beatriz Maestre-López, Marcelino González-Padilla, Pedro J. Carmona-Fernández, Antonio Vélez García-Nieto, and Beatriz Isla-Tejera

**Table.** AMSTAR checklist. The tool contains 11 questions (Q1-Q11) with regard to the quality of the review. Every question should be assigned a score 0 or 1. The sum of all scores is the overall methodological quality score of the systematic review.

| Item | Question | Responses | Code values |
| --- | --- | --- | --- |
| Q1 | Was an "a priori" design provided? | Yes[[1]](#footnote-2)/no/Can't answer/NA | 1/0/0/0 |
| Q2 | Was there duplicate study selection and data extraction? | Yes[[2]](#footnote-3)/no/Can't answer/NA | 1/0/0/0 |
| Q3 | Was a comprehensive literature search performed? | Yes[[3]](#footnote-4)/no/Can't answer/NA | 1/0/0/0 |
| Q4 | Was the status of publication (i.e. grey literature) used as an inclusion criterion? | Yes[[4]](#footnote-5)/no/Can't answer/NA | 1/0/0/0 |
| Q5 | Was a list of studies (included and excluded) provided? | Yes/no/Can't answer/NA | 1/0/0/0 |
| Q6 | Were the characteristics of the included studies provided? | Yes[[5]](#footnote-6)/no/Can't answer/NA | 1/0/0/0 |
| Q7 | Was the scientific quality of the included studies assessed and documented? | Yes[[6]](#footnote-7)/no/Can't answer/NA | 1/0/0/0 |
| Q8 | Was the scientific quality of the included studies used appropriately in formulating conclusions? | Yes[[7]](#footnote-8)/no/Can't answer/NA | 1/0/0/0 |
| Q9 | Were the methods used to combine the findings of studies appropriate? | Yes[[8]](#footnote-9)/no/Can't answer/NA | 1/0/0/0 |
| Q10 | Was the likelihood of publication bias assessed? | Yes[[9]](#footnote-10)/no/Can't answer/NA | 1/0/0/0 |
| Q11 | Was the conflict of interest included? | Yes[[10]](#footnote-11)/no/Can't answer/NA | 1/0/0/0 |

1. The research question and inclusion criteria were established before conducting the review. [↑](#footnote-ref-2)
2. At least two people working independently extracted the data and the method was reported for reaching consensus if disagreements arose. [↑](#footnote-ref-3)
3. At least two electronic sources were searched; details of the databases, years searched and search strategy were provided; the search was supplemented by searching of reference lists of included studies, and specialised registers, and by contacting experts. [↑](#footnote-ref-4)
4. The authors stated that they excluded studies from the review based on publication status. No−authors searched for reports irrespective of publication type. They did not exclude reports based on publication from the systematic review. [↑](#footnote-ref-5)
5. Data on participants, interventions and outcomes were provided, and the range of relevant characteristics reported. [↑](#footnote-ref-6)
6. Predetermined methods of assessing quality were reported. [↑](#footnote-ref-7)
7. The quality (and limitations) of included studies was used in the analysis, conclusions and recommendations of the review [↑](#footnote-ref-8)
8. If results were pooled statistically, heterogeneity was assessed and used to inform the decision of statistical model to be used. If heterogeneity was present, the appropriateness of combining studies was considered by review authors [↑](#footnote-ref-9)
9. Publication bias was explicitly considered and assessed. [↑](#footnote-ref-10)
10. Sources of support were clearly acknowledged. [↑](#footnote-ref-11)
