# Supplementary material for: Abstract analysis method facilitates filtering low-methodological quality and high-bias risk systematic reviews on psoriasis interventions
Source: BMC Med Res Methodol. 2017 Dec 29;17:180. doi: 10.1186/s12874-017-0460-z (PMC5747101; doi:10.1186/s12874-017-0460-z)
Supplement: Supplementary file 3 — Appendix 5. Phase 2 domains and signaling questions of ROBIS tool. (DOC 32 kb) [file 12874_2017_460_MOESM3_ESM.doc]

**Title**: Abstract analysis method facilitates filtering low-methodological quality and high-bias risk systematic reviews on psoriasis interventions

**Authors**: Francisco Gómez-García, Juan Ruano, Macarena Aguilar-Luque, Patricia Alcalde-Delgado, Jesús Gay-Mimbrera, José Luis Hernández-Romero, Juan Luis Sanz-Cabanillas, Beatriz Maestre-López, Marcelino González-Padilla, Pedro J. Carmona-Fernández, Antonio Vélez García-Nieto, and Beatriz Isla-Tejera

**Table.** Summary of phase 2 ROBIS domains and signaling questions.

| **Phase 2** | **Dominium** | **Signaling questions** | **Responses** | **Judgment** |
| --- | --- | --- | --- | --- |
|  | 1. Study elegibility criteria | 1.1. Did the review adhere to pre-defined objectives and eligibility criteria? | No/Probably no/Probably yes/Yes/No information | Concerns regarding specification of study elegibility criteria |
|  |  | 1.2. Were the eligibility criteria appropriate for the review question? | No/Probably no/Probably yes/Yes/No information |  |
|  |  | 1.3. Were eligibility criteria unambiguous? | No/Probably no/Probably yes/Yes/No information |  |
|  |  | 1.4. Were all restrictions in eligibility criteria based on study characteristics appropiate(e.g. date, sample size, study quality, outcomes measured)? | No/Probably no/Probably yes/Yes/No information |  |
|  |  | 1.5. Were any restrictions in eligibility criteria based on sources of information appropriate (e.g. publication status or format, language, availability of data)? | No/Probably no/Probably yes/Yes/No information |  |
|  | 2. Identification and selection of studies | 2.1. Did the search include an appropriate range of databases/electronic sources for published and unpublished reports? | No/Probably no/Probably yes/Yes/No information | Concerns regarding methods used to identify and/or select studies |
|  |  | 2.2. Were methods additional to database searching used to identify relevant reports? | No/Probably no/Probably yes/Yes/No information |  |
|  |  | 2.3. Were the terms and structure of the search strategy likely to retrieve as many eligible studies as possible? | No/Probably no/Probably yes/Yes/No information |  |
|  |  | 2.4. Were restrictions based on date, publication format, or language appropriate? | No/Probably no/Probably yes/Yes/No information |  |
|  |  | 2.5. Were efforts made to minimise error in selection of studies? | No/Probably no/Probably yes/Yes/No information |  |
|  | 3. Data collection and study appraisal | 3.1. Were efforts made to minimise error in data collection? | No/Probably no/Probably yes/Yes/No information | Concerns regarding methods used to collect data and appraise studies |
|  |  | 3.2. Were sufficient study characteristics available for both review authors and readers to be able to interpret the results? | No/Probably no/Probably yes/Yes/No information |  |
|  |  | 3.3. Were all relevant study results collected for use in the synthesis? | No/Probably no/Probably yes/Yes/No information |  |
|  |  | 3.4. Was risk of bias (or methodological quality) formally assessed using appropriate criteria? | No/Probably no/Probably yes/Yes/No information |  |
|  |  | 3.5. Were efforts made to minimise error in risk of bias assessment? | No/Probably no/Probably yes/Yes/No information |  |
|  | 4. Synthesis and findings | 4.1. Did the synthesis include all studies that it should? | No/Probably no/Probably yes/Yes/No information | Concerns regarding the synthesis |
|  |  | 4.2. Were all pre-defined analyses reported or departures explained? | No/Probably no/Probably yes/Yes/No information |  |
|  |  | 4.3. Was the synthesis appropriate given the nature and similarity in the research questions, study designs and outcomes across included studies? | No/Probably no/Probably yes/Yes/No information |  |
|  |  | 4.4. Was between-study variation (heterogeneity) minimal or addressed in the synthesis? | No/Probably no/Probably yes/Yes/No information |  |
|  |  | 4.5. Were the findings robust, e.g. as demonstrated through funnel plot or sensitivity analyses? | No/Probably no/Probably yes/Yes/No information |  |
|  |  | 4.6. Were biases in primary studies minimal or addressed in the synthesis? | No/Probably no/Probably yes/Yes/No information |  |
| Phase 3 |  | A. Did the interpretation of findings address all of the concerns identified in Domains 1 to 4? | No/Yes | Risk of bias in the review |
|  |  | B. Was the relevance of identified studies to the review's research question appropriately considered? | No/Yes |  |
|  |  | C. Did the reviewers avoid emphasizing results on the basis of their statistical significance? | No/Yes |  |
