# Supplementary material for: Abstract analysis method facilitates filtering low-methodological quality and high-bias risk systematic reviews on psoriasis interventions
Source: BMC Med Res Methodol. 2017 Dec 29;17:180. doi: 10.1186/s12874-017-0460-z (PMC5747101; doi:10.1186/s12874-017-0460-z)
Supplement: Supplementary file 4 — Appendix 2. List of included studies. (DOC 86 kb) [file 12874_2017_460_MOESM4_ESM.doc]

**Title**: Abstract analysis method facilitates filtering low-methodological quality and high-bias risk systematic reviews on psoriasis interventions

**Authors**: Francisco Gómez-García, Juan Ruano, Macarena Aguilar-Luque, Patricia Alcalde-Delgado, Jesús Gay-Mimbrera, José Luis Hernández-Romero, Juan Luis Sanz-Cabanillas, Beatriz Maestre-López, Marcelino González-Padilla, Pedro J. Carmona-Fernández, Antonio Vélez García-Nieto, and Beatriz Isla-Tejera

**List of included studies.**

**2017**

1. Ali FM, Cueva AC, Vyas J, Atwan AA, Salek MS, Finlay AY, Piguet V. A systematic review of the use of quality-of-life instruments in randomized controlled trials for psoriasis. Br J Dermatol 2017;176:577-93. doi: 10.1111/bjd.14788.
2. Brown G, Wang E, Leon A, Huynh M, Wehner M, Matro R, Linos E, Liao W, Haemel A. Tumor necrosis factor-α inhibitor-induced psoriasis: Systematic review of clinical features, histopathological findings, and management experience. J Am Acad Dermatol 2017;76:334-341. doi: 10.1016/j.jaad.2016.08.012.
3. de Carvalho AV, Duquia RP, Horta BL, Bonamigo RR. Efficacy of Immunobiologic and Small Molecule Inhibitor Drugs for Psoriasis: A Systematic Review and Meta-Analysis of Randomized Clinical Trials. Drugs R D 2017;17:29-51.
4. Gómez-García F, Epstein D, Isla-Tejera B, Lorente A, Vélez García-Nieto A, Ruano J. Short-term efficacy and safety of new biological agents targeting the interleukin-23-T helper 17 pathway for moderate-to-severe plaque psoriasis: a systematic review and network meta-analysis. Br J Dermatol 2017;176:594-603. doi: 10.1111/bjd.14814.
5. Puig L, Thom H, Mollon P, Tian H, Ramakrishna GS. Clear or almost clear skin improves the quality of life in patients with moderate-to-severe psoriasis: a systematic review and meta-analysis. J Eur Acad Dermatol Venereol 2017;31:213-220. doi: 10.1111/jdv.14007.

**2016**

1. Belinchón I, Rivera R, Blanch C, Comellas M, Lizán L. Adherence, satisfaction and preferences for treatment in patients with psoriasis in the European Union: a systematic review of the literature. Patient Prefer Adherence 2016 Nov;10:2357-2367. ECollection 2016.
2. Chen HQ, Li X, Tang R. Effects of Narrow Band Ultraviolet B on Serum Levels of Vascular Endothelial Growth Factor and Interleukin-8 in Patients with Psoriasis Am J Ther 2016;23:e655-62. doi: 10.1097/MJT.0000000000000330.
3. de Carvalho AV, Duquia RP, Horta BL, Bonamigo RR. Efficacy of Immunobiologic and Small Molecule Inhibitor Drugs for Psoriasis: A Systematic Review and Meta-Analysis of Randomized Clinical Trials. Drugs R D 2017;17:29-51. doi: 10.1007/s40268-016-0152-x.
4. De Vecchis R, Baldi C, Palmisani L. Protective effects of methotrexate against ischemic cardiovascular disorders in patients treated for rheumatoid arthritis or psoriasis: novel therapeutic insights coming from a meta-analysis of the literature data. Anatol J Cardiol 2016;16:2-9. doi: 10.5152/akd.2015.6136.
5. Desai RJ, Thaler KJ, Mahlknecht P, Gartlehner G, McDonagh MS, Mesgarpour B, Mazinanian A, Glechner A, Gopalakrishnan C, Hansen RA. Comparative Risk of Harm Associated With the Use of Targeted Immunomodulators: A Systematic Review. Arthritis Care Res (Hoboken) 2016;68:1078-88. doi: 10.1002/acr.22815.
6. Gutknecht M, Schaarschmidt ML, Herrlein O, Augustin M. A systematic review on methods used to evaluate patient preferences in psoriasis treatments. J Eur Acad Dermatol Venereol 2016;30:1454-64. doi: 10.1111/jdv.13749.
7. Jacobs I, Petersel D, Isakov L, Lula S, Lea Sewell K. Biosimilars for the Treatment of Chronic Inflammatory Diseases: A Systematic Review of Published Evidence. BioDrugs 2016;30:525-70.
8. Obradors M, Blanch C, Comellas M, Figueras M, Lizan L. Health-related quality of life in patients with psoriasis: a systematic review of the European literature. Qual Life Res 2016;25:2739-54.
9. Pickett K, Frampton G, Loveman E. Education to improve quality of life of people with chronic inflammatory skin conditions: a systematic review of the evidence. British Journal of Dermatology 2016;174:1228-41.
10. Ryoo JY, Yang HJ, Ji E, Yoo BK. Meta-analysis of the Efficacy and Safety of Secukinumab for the Treatment of Plaque Psoriasis. Ann Pharmacother 2016;50:341-51. doi: 10.1177/1060028015626545.
11. Schlager JG, Rosumeck S, Werner RN, Jacobs A, Schmitt J, Schlager C, et al. Topical treatments for scalp psoriasisCochrane Database of Systematic Reviews 2016:2:CD009687.
12. Shelton E, Laharie D, Scott FI, Mamtani R, Lewis JD, Colombel JF, Ananthakrishnan AN. Cancer Recurrence Following Immune-Suppressive Therapies in Patients With Immune-Mediated Diseases: A Systematic Review and Meta-analysis. Gastroenterology 2016;151:97-109.e4. doi: 10.1053/j.gastro.2016.03.037.
13. Svendsen MT, Jeyabalan J, Andersen KE, Andersen F, Johannessen H. Worldwide utilization of topical remedies in treatment of psoriasis: a systematic review. J Dermatolog Treat 2016;13:1-10.
14. Teixeira A, Teixeira M, Almeida V, Torres T, Sousa Lobo JM, Almeida IF. Methodologies for medication adherence evaluation: Focus on psoriasis topical treatment. Journal of Dermatological Science 2016;82:63-8.
15. Vaughn AR, Branum A, Sivamani RK. Effects of Turmeric (Curcuma longa) on Skin Health: A Systematic Review of the Clinical Evidence. Phytother Res 2016;30:1243-64. doi: 10.1002/ptr.5640.
16. Wang J, Zhan Q, Zhang L. A systematic review on the efficacy and safety of Infliximab in patients with psoriasis. Hum Vaccin Immunother 2016;12:431-37.
17. West J, Ogston S, Foerster J. Safety and efficacy of methotrexate in psoriasis: A meta-analysis of published trials. PLoS ONE 2016;11:5: e0153740.
18. Yamauchi PS, Bissonnette R, Teixeira HD, Valdecantos WC. Systematic review of efficacy of anti-tumor necrosis factor (TNF) therapy in patients with psoriasis previously treated with a different anti-TNF agent. J Am Acad Dermatol 2016;75:612-618.e6. doi: 10.1016/j.jaad.2016.02.1221.
19. Yan R, Jiang S, Wu Y, Gao XH, Chen HD. Topical calcipotriol/betamethasone dipropionate for psoriasis vulgaris: A systematic review.Indian Journal of Dermatology, Venereology and Leprology 2016;82:135-44.
20. Yang ZS, Lin NN, Li L, Li Y. The Effect of TNF Inhibitors on Cardiovascular Events in Psoriasis and Psoriatic Arthritis: an Updated Meta-Analysis Clinical Reviews in Allergy and Immunology 2016;(1-8).
21. Yiu ZZ, Exton LS, Jabbar-Lopez Z, Mohd Mustapa MF, Samarasekera EJ, Burden AD, Murphy R, Owen CM, Parslew R, Venning V, Ashcroft DM, Griffiths CE, Smith CH, Warren RB. Risk of Serious Infections in Patients with Psoriasis on Biologic Therapies: A Systematic Review and Meta-Analysis. J Invest Dermatol 2016;136:1584-91. doi: 10.1016/j.jid.2016.03.035.
22. Zhang CS, Yang L, Zhang AL, May BH, Yu JJ, Guo X, et al. Is Oral Chinese Herbal Medicine Beneficial for Psoriasis Vulgaris? A Meta-Analysis of Comparisons with Acitretin Journal of Alternative and Complementary Medicine 2016;22:174-88.

**2015**

1. Almutawa F, Thalib L, Hekman D, Sun Q, Hamzavi I, Lim HW. Efficacy of localized phototherapy and photodynamic therapy for psoriasis: a systematic review and meta-analysis. Photodermatol Photoimmunol Photomed 2015;31:5-14. doi: 10.1111/phpp.12092.
2. Atwan A, Ingram JR, Abbott R, Kelson MJ, Pickles T, Bauer A, Piguet V. Oral fumaric acid esters for psoriasis: abridged Cochrane systematic review including GRADE assessments. Br J Dermatol 2016;175:873-881. doi: 10.1111/bjd.14676.
3. Chen Y, Qian T, Zhang D, Yan H, Hao F. Clinical efficacy and safety of anti-IL-17 agents for the treatment of patients with psoriasis. Immunotherapy 2015;7:1023-37. doi: 10.2217/imt.15.50.
4. Conway R, Low C, Coughlan RJ, O'Donnell MJ, Carey JJ. Methotrexate use and risk of lung disease in psoriasis, psoriatic arthritis, and inflammatory bowel disease: systematic literature review and meta-analysis of randomised controlled trials. BMJ 2015;13:h1269. doi: 10.1136/bmj.h1269.
5. Coyle M, Deng J, Zhang AL, Yu J, Guo X, Xue CC, et al. Acupuncture therapies for psoriasis vulgaris: a systematic review of randomized controlled trials. Forsch Komplementmed 2015;22:102-9. doi: 10.1159/000381225.
6. Dannepond C, Maruani A, Machet L, Ternant D, Paintaud G, Samimi M. Serum infliximab concentrations in psoriatic patients treated with infliximab: a systematic review. Acta Derm Venereol 2015;95:401-6. doi: 10.2340/00015555-1980.
7. Depont F, Berenbaum F, Filippi J, Le Maitre M, Nataf H, Paul C, Peyrin-Biroulet L, Thibout E. Interventions to Improve Adherence in Patients with Immune-Mediated Inflammatory Disorders: A Systematic Review. PLoS One 2015;10:e0145076. doi: 10.1371/journal.pone.0145076. ECollection 2015.
8. Fleming P, Roubille C, Richer V, Starnino T, McCourt C, McFarlane A, et al. Effect of biologics on depressive symptoms in patients with psoriasis: a systematic review. J Eur Acad Dermatol Venereol 2015;29:1063-70. doi: 10.1111/jdv.12909.
9. Jacobi A, Mayer A, Augustin M. Keratolytics and emollients and their role in the therapy of psoriasis: a systematic review. Dermatol Ther (Heidelb) 2015;5(1):1-18.
10. Jacobs A, Rosumeck S, Nast A. Systematic review on the maintenance of response during systemic antipsoriatic therapy. Br J Dermatol 2015;173:910-21. doi: 10.1111/bjd.14077.
11. Miroddi M, Navarra M, Calapai F, Mancari F, Giofrè SV, Gangemi S, Calapai G. Review of Clinical Pharmacology of Aloe vera L. in the Treatment of Psoriasis. Phytother Res 2015;29:648-55. doi: 10.1002/ptr.5316
12. Nast A, Jacobs A, Rosumeck S, Werner RN. Efficacy and Safety of Systemic Long-Term Treatments for Moderate-to-Severe Psoriasis: A Systematic Review and Meta-Analysis. J Invest Dermatol 2015;135:2641-8. doi: 10.1038/jid.2015.206.
13. Nast A, Rosumeck S, Seidenschnur K. Biosimilars: a systematic review of published and ongoing clinical trials of antipsoriatics in chronic inflammatory diseases. J Dtsch Dermatol Ges 2015;13:294-300. doi: 10.1111/ddg.12621.
14. Pickett K, Loveman E, Kalita N, Frampton GK, Jones J. Educational interventions to improve quality of life in people with chronic inflammatory skin diseases: systematic reviews of clinical effectiveness and cost-effectiveness. Health Technol Assess 2015;19:1-176, v-vi. doi: 10.3310/hta19860.
15. Rachakonda TD, Dhillon JS, Florek AG, Armstrong AW. Effect of tonsillectomy on psoriasis: a systematic review. J Am Acad Dermatol 2015;72:261-75.
16. Roubille C, Richer V, Starnino T, McCourt C, McFarlane A, Fleming P, et al. The effects of tumour necrosis factor inhibitors, methotrexate, non-steroidal anti-inflammatory drugs and corticosteroids on cardiovascular events in rheumatoid arthritis, psoriasis and psoriatic arthritis: a systematic review and meta-analysis. Ann Rheum Dis 2015;74:480-9. doi: 10.1136/annrheumdis-2014-206624.
17. Sanclemente G, Murphy R, Contreras J, García H, Bonfill Cosp X. Anti-TNF agents for paediatric psoriasis. Cochrane Database Syst Rev. 2015:CD010017. doi: 10.1002/14651858.CD010017.pub2.
18. Signorovitch JE, Betts KA, Yan YS, LeReun C, Sundaram M, Wu EQ, et al. Comparative efficacy of biological treatments for moderate-to-severe psoriasis: a network meta-analysis adjusting for cross-trial differences in reference arm response. Br J Dermatol 2015;172:504-12. doi: 10.1111/bjd.13437.
19. Upala S, Sanguankeo A. Effect of lifestyle weight loss intervention on disease severity in patients with psoriasis: a systematic review and meta-analysis. Int J Obes (Lond). 2015;39:1197-202. doi: 10.1038/ijo.2015.64.
20. Vangeli E, Bakhshi S, Baker A, Fisher A, Bucknor D, Mrowietz U, Östör AJ, Peyrin-Biroulet L, Lacerda AP, Weinman J. A Systematic Review of Factors Associated with Non-Adherence to Treatment for Immune-Mediated Inflammatory Diseases. Adv Ther 2015;32:983-1028. doi: 10.1007/s12325-015-0256-7.
21. Yang L, Zhang CS, May B, Yu J, Guo X, Zhang AL, Xue CC, Lu C. Efficacy of combining oral Chinese herbal medicine and NB-UVB in treating psoriasis vulgaris: a systematic review and meta-analysis. Chin Med 2015;10:27. doi: 10.1186/s13020-015-0060-y. eCollection 2015.

**2014**

1. Augustin M, Mrowietz U, Bonnekoh B, Rosenbach T, Thaçi D, Reusch M, et al. Topical long-term therapy of psoriasis with vitamin D₃ analogues, corticosteroids and their two compound formulations: position paper on evidence and use in daily practice. J Dtsch Dermatol Ges 2014;12:667-82. doi: 10.1111/ddg.12396.
2. Boehncke WH, Alvarez Martinez D, Solomon JA, Gottlieb AB. Safety and efficacy of therapies for skin symptoms of psoriasis in patients with psoriatic arthritis: a systematic review. J Rheumatol 2014;41:2301-5. doi: 10.3899/jrheum.140880
3. Busard C, Zweegers J, Limpens J, Langendam M, Spuls PI. Combined use of systemic agents for psoriasis: a systematic review. JAMA Dermatol 2014;150:1213-20. Doi: 10.1001/jamadermatol.2014.1111.
4. Deng S, May BH, Zhang AL, Lu C, Xue CC. Topical herbal formulae in the management of psoriasis: systematic review with meta-analysis of clinical studies and investigation of the pharmacological actions of the main herbs. Phytother Res 2014;28:480-97. doi: 10.1002/ptr.5028.
5. Gupta AK, Daigle D, Lyons DC. Network Meta-analysis of Treatments for Chronic Plaque Psoriasis in Canada. J Cutan Med Surg 2014;18:371-8.
6. Hsu L, Snodgrass BT, Armstrong AW. Antidrug antibodies in psoriasis: a systematic review. Br J Dermatol 2014;170:261-73. doi: 10.1111/bjd.12654.
7. Larsen MH, Hagen KB, Krogstad AL, Aas E, Wahl AK. Limited evidence of the effects of patient education and self-management interventions in psoriasis patients: a systematic review. Patient Educ Couns 2014;94:158-69. doi: 10.1016/j.pec.2013.10.005.
8. Mattei PL, Corey KC, Kimball AB. Psoriasis Area Severity Index (PASI) and the Dermatology Life Quality Index (DLQI): the correlation between disease severity and psychological burden in patients treated with biological therapies. J Eur Acad Dermatol Venereol 2014;28:333-7. doi: 10.1111/jdv.12106.
9. Maybury CM, Jabbar-Lopez ZK, Wong T, Dhillon AP, Barker JN, Smith CH. Methotrexate and liver fibrosis in people with psoriasis: a systematic review of observational studies. Br J Dermatol 2014;171:17-29. doi: 10.1111/bjd.12941.
10. Meng Y, Dongmei L, Yanbin P, Jinju F, Meile T, Binzhu L, et al. Systematic review and meta-analysis of ustekinumab for moderate to severe psoriasis. Clin Exp Dermatol 2014;39:696-707. doi: 10.1111/ced.12390
11. Posso-De Los Rios CJ, Pope E, Lara-Corrales I. A systematic review of systemic medications for pustular psoriasis in pediatrics. Pediatr Dermatol 2014;31:430-9. doi: 10.1111/pde.12351.
12. Puig L, López A, Vilarrasa E, García I. Efficacy of biologics in the treatment of moderate-to-severe plaque psoriasis: a systematic review and meta-analysis of randomized controlled trials with different time points. J Eur Acad Dermatol Venereol 2014;28:1633-53. doi: 10.1111/jdv.12238.
13. Sandoval LF, Pierce A, Feldman SR. Systemic therapies for psoriasis: an evidence-based update. Am J Clin Dermatol 2014;15:165-80. doi: 10.1007/s40257-014-0064-x.
14. Schmitt J, Rosumeck S, Thomaschewski G, Sporbeck B, Haufe E, Nast A. Efficacy and safety of systemic treatments for moderate-to-severe psoriasis: meta-analysis of randomized controlled trials.Br J Dermatol 2014;170:274-303. doi: 10.1111/bjd.12663.
15. Sevrain M, Richard MA, Barnetche T, Rouzaud M, Villani AP, Paul C, et al. Treatment for palmoplantar pustular psoriasis: systematic literature review, evidence-based recommendations and expert opinion. J Eur Acad Dermatol Venereol 2014;28 Suppl 5:13-6. doi: 10.1111/jdv.12561.
16. Zhang CS, Yu JJ, Parker S, Zhang AL, May B, Lu C, et al. Oral Chinese herbal medicine combined with pharmacotherapy for psoriasis vulgaris: a systematic review. Int J Dermatol 2014;53:1305-18. doi: 10.1111/ijd.12607.
17. Zhou D, Chen W, Li X, et al. Evidence-based practice guideline of Chinese herbal medicine for psoriasis vulgaris (Bai Bi) Eur J Integr Med 2014;6:135-46.

**2013**

1. Almutawa F, Alnomair N, Wang Y, Hamzavi I, Lim HW. Systematic review of UV-based therapy for psoriasis. Am J Clin Dermatol 2013;14:87-109. doi: 10.1007/s40257-013-0015-y.
2. Chen X, Yang M, Cheng Y, Liu GJ, Zhang M. Narrow-band ultraviolet B phototherapy versus broad-band ultraviolet B or psoralen-ultraviolet A photochemotherapy for psoriasis. Cochrane Database Syst Rev 2013:CD009481. doi: 10.1002/14651858.CD009481.pub2.
3. Correr CJ, Rotta I, Teles T de S, Godoy RR, Riveros BS, Garcia MM, et al. Efficacy and safety of biologics in the treatment of moderate to severe psoriasis: a comprehensive meta-analysis of randomized controlled trials. Cad Saude Publica 2013;29 Suppl 1:S17-31.
4. de Vries AC, Bogaards NA, Hooft L, Velema M, Pasch M, Lebwohl M, Spuls PI. Interventions for nail psoriasis. Cochrane Database Syst Rev 2013;31:CD007633. doi: 10.1002/14651858.CD007633.pub2.
5. Deng S, May BH, Zhang AL, Lu C, Xue CC. Plant extracts for the topical management of psoriasis: a systematic review and meta-analysis. Br J Dermatol 2013;169:769-82. doi: 10.1111/bjd.12557.
6. Deng S, May BH, Zhang AL, Lu C, Xue CC. Plant extracts for the topical management of psoriasis: a systematic review and meta-analysis. Br J Dermatol 2013;169:769-82. doi: 10.1111/bjd.12557.
7. Deng S, May BH, Zhang AL, Lu C, Xue CC. Topical herbal medicine combined with pharmacotherapy for psoriasis: a systematic review and meta-analysis. Arch Dermatol Res 2013 Apr;305(3):179-89. doi: 10.1007/s00403-013-1316-y.
8. Erceg A, de Jong EM, van de Kerkhof PC, Seyger MM. The efficacy of pulsed dye laser treatment for inflammatory skin diseases: a systematic review. J Am Acad Dermatol 2013;69:609-615.e8. doi: 10.1016/j.jaad.2013.03.029.
9. Galván-Banqueri M, Marín Gil R, Santos Ramos B, Bautista Paloma FJ. Biological treatments for moderate-to-severe psoriasis: indirect comparison. J Clin Pharm Ther 2013;38:121-30. doi: 10.1111/jcpt.12044.
10. Garcês S, Demengeot J, Benito-Garcia E. The immunogenicity of anti-TNF therapy in immune-mediated inflammatory diseases: a systematic review of the literature with a meta-analysis. Ann Rheum Dis 2013;72:1947-55. doi: 10.1136/annrheumdis-2012-202220.
11. Hendriks AG, Keijsers RR, de Jong EM, Seyger MM, van de Kerkhof PC. Combinations of classical time-honoured topicals in plaque psoriasis: a systematic review. J Eur Acad Dermatol Venereol 2013;27:399-410. doi: 10.1111/j.1468-3083.2012.04640.x.
12. Hendriks AG, Keijsers RR, de Jong EM, Seyger MM, van de Kerkhof PC. Efficacy and safety of combinations of first-line topical treatments in chronic plaque psoriasis: a systematic literature review. J Eur Acad Dermatol Venereol 2013;27:931-51. doi: 10.1111/jdv.12058.
13. Maneiro JR, Salgado E, Gomez-Reino JJ. Immunogenicity of monoclonal antibodies against tumor necrosis factor used in chronic immune-mediated Inflammatory conditions: systematic review and meta-analysis. JAMA Intern Med 2013;12;173:1416-28. doi: 10.1001/jamainternmed.2013.7430.
14. Mason A, Mason J, Cork M, Hancock H, Dooley G. Topical treatments for chronic plaque psoriasis: an abridged Cochrane systematic review. J Am Acad Dermatol 2013;69:799-807. doi: 10.1016/j.jaad.2013.06.027.
15. Mason AR, Mason J, Cork M, Dooley G, Hancock H. Topical treatments for chronic plaque psoriasis. Cochrane Database Syst Rev 2013; 28:CD005028. doi: 10.1002/14651858.CD005028.pub3.
16. Mason AR, Mason JM, Cork MJ, Hancock H, Dooley G. Topical treatments for chronic plaque psoriasis of the scalp: a systematic review. Br J Dermatol 2013;169:519-27. doi: 10.1111/bjd.12393.
17. Mustafa AA, Al-Hoqail IA. Biologic systemic therapy for moderate-to-severe psoriasis: A review. Journal of Taibah University Medical Sciences 2013;8:142-50.
18. Samarasekera EJ, Sawyer L, Wonderling D, Tucker R, Smith CH. Topical therapies for the treatment of plaque psoriasis: systematic review and network meta-analyses. Br J Dermatol 2013 May;168(5):954-67. doi: 10.1111/bjd.12276.
19. Strohal R, Chimenti S, Vena GA, Girolomoni G. Etanercept provides an effective, safe and flexible short- and long-term treatment regimen for moderate-to-severe psoriasis: a systematic review of current evidence. J Dermatolog Treat 2013;24:199-208. doi: 10.3109/09546634.2012.713462.
20. Thorlund K, Druyts E, Aviña-Zubieta JA, Mills EJ. Anti-tumor necrosis factor (TNF) drugs for the treatment of psoriatic arthritis: an indirect comparison meta-analysis. Biologics 2012;6:417-27. doi: 10.2147/BTT.S37606.
21. Thorneloe RJ, Bundy C, Griffiths CE, Ashcroft DM, Cordingley L. Adherence to medication in patients with psoriasis: a systematic literature review. Br J Dermatol 2013;168:20-31. doi: 10.1111/bjd.12039.
22. Yu JJ, Zhang CS, Zhang AL, May B, Xue CC, Lu C. Add-on effect of chinese herbal medicine bath to phototherapy for psoriasis vulgaris: a systematic review. Evid Based Complement Alternat Med 2013:673078. doi: 10.1155/2013/673078

**2012**

1. Archier E, Devaux S, Castela E, Gallini A, Aubin F, Le Maître M, et al. Carcinogenic risks of psoralen UV-A therapy and narrowband UV-B therapy in chronic plaque psoriasis: a systematic literature review. J Eur Acad Dermatol Venereol 2012;26 Suppl 3:22-31. doi: 10.1111/j.1468-3083.2012.04520.x.
2. Archier E, Devaux S, Castela E, Gallini A, Aubin F, Le Maître M, et al. Efficacy of psoralen UV-A therapy vs. narrowband UV-B therapy in chronic plaque psoriasis: a systematic literature review. J Eur Acad Dermatol Venereol 2012;26 Suppl 3:11-21. doi: 10.1111/j.1468-3083.2012.04519.x.
3. Archier E, Devaux S, Castela E, Gallini A, Aubin F, Le Maître M, et al. Ocular damage in patients with psoriasis treated by psoralen UV-A therapy or narrow band UVB therapy: a systematic literature review. J Eur Acad Dermatol Venereol 2012;26 Suppl 3:32-5. doi: 10.1111/j.1468-3083.2012.04521.x.
4. Bailey EE, Ference EH, Alikhan A, Hession MT, Armstrong AW. Combination treatments for psoriasis: a systematic review and meta-analysis. Arch Dermatol 2012;148:511-22. doi: 10.1001/archdermatol.2011.1916.
5. Brezinski EA, Armstrong AW. Off-label biologic regimens in psoriasis: a systematic review of efficacy and safety of dose escalation, reduction, and interrupted biologic therapy. PLoS One. 2012;7(4):e33486. doi: 10.1371/journal.pone.0033486. 1.
6. Castela E, Archier E, Devaux S, Gallini A, Aractingi S, Cribier B, et al. Topical corticosteroids in plaque psoriasis: a systematic review of efficacy and treatment modalities. J Eur Acad Dermatol Venereol 2012;26 Suppl 3:36-46. doi: 10.1111/j.1468-3083.2012.04522.x.
7. Castela E, Archier E, Devaux S, Gallini A, Aractingi S, Cribier B, et al. Topical corticosteroids in plaque psoriasis: a systematic review of risk of adrenal axis suppression and skin atrophy. J Eur Acad Dermatol Venereol 2012;26 Suppl 3:47-51. doi: 10.1111/j.1468-3083.2012.04523.x.
8. Devaux S, Castela A, Archier E, Gallini A, Joly P, Misery L, Aractingi S, Aubin F, Bachelez H, Cribier B, Jullien D, Le Maître M, Richard MA, Ortonne JP, Paul C. Adherence to topical treatment in psoriasis: a systematic literature review. J Eur Acad Dermatol Venereol 2012;26 Suppl 3:61-7. doi: 10.1111/j.1468-3083.2012.04525.x. Review.
9. Devaux S, Castela A, Archier E, Gallini A, Joly P, Misery L, et al. Topical vitamin D analogues alone or in association with topical steroids for psoriasis: a systematic review. J Eur Acad Dermatol Venereol 2012;Suppl 3:52-60. doi: 10.1111/j.1468-3083.2012.04524.x.
10. Kim IH, West CE, Kwatra SG, Feldman SR, O'Neill JL. Comparative efficacy of biologics in psoriasis: a review. Am J Clin Dermatol 2012;13:365-74. doi: 10.2165/11633110-000000000-00000.
11. Lamel SA, Myer KA, Younes N, Zhou JA, Maibach H, Maibach HI. Placebo response in relation to clinical trial design: a systematic review and meta-analysis of randomized controlled trials for determining biologic efficacy in psoriasis treatment. Arch Dermatol Res 2012;304:707-17. doi: 10.1007/s00403-012-1266-9.
12. Li N, Li YQ, Li HY, Guo W, Bai YP. Efficacy of externally applied Chinese herbal drugs in treating psoriasis: a systematic review. Chin J Integr Med 2012;18(3):222-9. doi: 10.1007/s11655-012-1004-3.
13. Lin VW, Ringold S, Devine EB. Comparison of Ustekinumab With Other Biological Agents for the Treatment of Moderate to Severe Plaque Psoriasis: A Bayesian Network Meta-analysis. Arch Dermatol 2012;148:1403-10. doi: 10.1001/2013.jamadermatol.238.
14. Lucka TC, Pathirana D, Sammain A, Bachmann F, Rosumeck S, Erdmann R, et al. Efficacy of systemic therapies for moderate-to-severe psoriasis: a systematic review and meta-analysis of long-term treatment. J Eur Acad Dermatol Venereol 2012;26:1331-44. doi: 10.1111/j.1468-3083.2012.04492.x.
15. Malhotra A, Shafiq N, Rajagopalan S, Dogra S, Malhotra S. Thiazolidinediones for plaque psoriasis: a systematic review and meta-analysis. Evid Based Med. 2012;17:171-6. doi: 10.1136/ebmed-2011-100388.
16. Paul C, Gallini A, Archier E, Castela E, Devaux S, Aractingi S, et al. Evidence-based recommendations on topical treatment and phototherapy of psoriasis: systematic review and expert opinion of a panel of dermatologists. J Eur Acad Dermatol Venereol 2012;26 Suppl 3:1-10. doi: 10.1111/j.1468-3083.2012.04518.x.
17. Reich K, Burden AD, Eaton JN, Hawkins NS. Efficacy of biologics in the treatment of moderate to severe psoriasis: a network meta-analysis of randomized controlled trials. Br J Dermatol 2012;166(1):179-88. doi: 10.1111/j.1365-2133.2011.10583.x.
18. Umar N, Yamamoto S, Loerbroks A, Terris D. Elicitation and use of patients' preferences in the treatment of psoriasis: a systematic review. Acta Derm Venereol 2012;92:341-6. doi: 10.2340/00015555-1304.

**2011**

1. Atwan A, Ingram JR, Abbott R, Kelson MJ, Pickles T, Bauer A, Piguet V. Oral fumaric acid esters for psoriasis: abridged Cochrane systematic review including GRADE assessments. Br J Dermatol 2016;175:873-81. doi: 10.1111/bjd.14676.
2. Bottomley JM, Taylor RS, Ryttov J. The effectiveness of two-compound formulation calcipotriol and betamethasone dipropionate gel in the treatment of moderately severe scalp psoriasis: a systematic review of direct and indirect evidence. Curr Med Res Opin 2011;27:251-68. doi: 10.1185/03007995.2010.541022.
3. Dommasch ED, Abuabara K, Shin DB, Nguyen J, Troxel AB, Gelfand JM. The risk of infection and malignancy with tumor necrosis factor antagonists in adults with psoriatic disease: a systematic review and meta-analysis of randomized controlled trials. J Am Acad Dermatol 2011;64:1035-50. doi: 10.1016/j.jaad.2010.09.734.
4. Langham S, Langham J, Goertz HP, Ratcliffe M. Large-scale, prospective, observational studies in patients with psoriasis and psoriatic arthritis: A systematic and critical review. BMC Med Res Methodol 2011;11:32. doi: 10.1186/1471-2288-11-32.
5. Montaudié H, Sbidian E, Paul C, Maza A, Gallini A, Aractingi S, et al. Methotrexate in psoriasis: a systematic review of treatment modalities, incidence, risk factors and monitoring of liver toxicity. J Eur Acad Dermatol Venereol 2011;25 Suppl 2:12-8. doi: 10.1111/j.1468-3083.2011.03991.x.
6. Ryan C, Leonardi CL, Krueger JG, Kimball AB, Strober BE, Gordon KB, et al. Association between biologic therapies for chronic plaque psoriasis and cardiovascular events: a meta-analysis of randomized controlled trials. JAMA 2011;306:864-71. doi: 10.1001/jama.2011.1211.
7. Maza A, Montaudié H, Sbidian E, Gallini A, Aractingi S, Aubin F, et al. Oral cyclosporin in psoriasis: a systematic review on treatment modalities, risk of kidney toxicity and evidence for use in non-plaque psoriasis. J Eur Acad Dermatol Venereol 2011;25 Suppl 2:19-27. doi: 10.1111/j.1468-3083.2011.03992.x.
8. Sbidian E, Maza A, Montaudié H, Gallini A, Aractingi S, Aubin F, et al. Efficacy and safety of oral retinoids in different psoriasis subtypes: a systematic literature review. J Eur Acad Dermatol Venereol 2011;25 Suppl 2:28-33. doi: 10.1111/j.1468-3083.2011.03993.x.
9. Tan JY, Li S, Yang K, Ma B, Chen W, Zha C, Zhang J. Ustekinumab, a human interleukin-12/23 monoclonal antibody, in patients with psoriasis: a meta-analysis. J Dermatolog Treat 2011;22:323-36. Doi: 10.3109/09546634.2010.487890.

**2010**

1. de Jager ME, de Jong EM, van de Kerkhof PC, Seyger MM. Efficacy and safety of treatments for childhood psoriasis: a systematic literature review. J Am Acad Dermatol 2010;62:1013-30. doi: 10.1016/j.jaad.2009.06.048.

**2009**

1. Bansback N, Sizto S, Sun H, Feldman S, Willian MK, Anis A. Efficacy of systemic treatments for moderate to severe plaque psoriasis: systematic review and meta-analysis. Dermatology 2009;219:209-18. doi: 10.1159/000233234.
2. Feldman SR, Yentzer BA. Topical clobetasol propionate in the treatment of psoriasis: a review of newer formulations. Am J Clin Dermatol 2009;10:397-406. doi: 10.2165/11311020-000000000-00000.
3. Naldi L, Rzany B. Psoriasis (chronic plaque). BMJ Clin Evid 2009. pii: 1706.
4. Smith N, Weymann A, Tausk FA, Gelfand JM. Complementary and alternative medicine for psoriasis: a qualitative review of the clinical trial literature. J Am Acad Dermatol 2009;61(5):841-56. doi: 10.1016/j.jaad.2009.04.029.
5. Prey S, Paul C. Effect of folic or folinic acid supplementation on methotrexate-associated safety and efficacy in inflammatory disease: a systematic review. Br J Dermatol 2009;160:622-8. doi: 10.1111/j.1365-2133.2008.08876.x.

**2008**

1. Brimhall AK, King LN, Licciardone JC, Jacobe H, Menter A. Safety and efficacy of alefacept, efalizumab, etanercept and infliximab in treating moderate to severe plaque psoriasis: a meta-analysis of randomized controlled trials. Br J Dermatol 2008;159:274-85. doi: 10.1111/j.1365-2133.2008.08673.x.
2. Schmitt J, Zhang Z, Wozel G, Meurer M, Kirch W. Efficacy and tolerability of biologic and nonbiologic systemic treatments for moderate-to-severe psoriasis: meta-analysis of randomized controlled trials. Br J Dermatol 2008;159:513-26. doi: 10.1111/j.1365-2133.2008.08732.x.

**2006**

1. Boehncke WH, Prinz J, Gottlieb AB. Biologic therapies for psoriasis. A systematic review. J Rheumatol 2006;33:1447-51.
2. Cassell S, Kavanaugh AF. Therapies for psoriatic nail disease. A systematic review. J Rheumatol 2006;33:1452-6.
3. Marsland AM, Chalmers RJ, Hollis S, Leonardi-Bee J, Griffiths CE. Interventions for chronic palmoplantar pustulosis. Cochrane Database Syst Rev 2006:CD001433.
4. Strober BE, Siu K, Menon K. Conventional systemic agents for psoriasis. A systematic review. J Rheumatol 2006;33:1442-6.
5. Woolacott N, Hawkins N, Mason A, Kainth A, Khadjesari Z, Vergel YB, Misso K, Light K, Chalmers R, Sculpher M, Riemsma R. Etanercept and efalizumab for the treatment of psoriasis: a systematic review. Health Technol Assess. 2006;10:1-233, i-iv.

**2003**

1. Bruner CR, Feldman SR, Ventrapragada M, Fleischer AB Jr. A systematic review of adverse effects associated with topical treatments for psoriasis. Dermatol Online J 2003;9:2.

**2002**

1. Heydendael VM, Spuls PI, Ten Berge IJ, Opmeer BC, Bos JD, de Rie MA. Cyclosporin trough levels: is monitoring necessary during short-term treatment in psoriasis? A systematic review and clinical data on trough levels. Br J Dermatol 2002;147:122-9.
2. Mason J, Mason AR, Cork MJ. Topical preparations for the treatment of psoriasis: a systematic review. Br J Dermatol 2002;146:351-64.

**2001**

1. Chalmers RJ, O'Sullivan T, Owen CM, Griffiths CE. A systematic review of treatments for guttate psoriasis. Br J Dermatol 2001;145:891-4.
2. Owen CM, Chalmers RJ, O'Sullivan T, Griffiths CE. A systematic review of antistreptococcal interventions for guttate and chronic plaque psoriasis. Br J Dermatol 2001;145:886-90.

**2000**

1. Ashcroft DM, Li Wan Po A, Williams HC, Griffiths CE. Combination regimens of topical calcipotriene in chronic plaque psoriasis: systematic review of efficacy and tolerability. Arch Dermatol 2000;136:1536-43.
2. Ashcroft DM, Po AL, Williams HC, Griffiths CE. Systematic review of comparative efficacy and tolerability of calcipotriol in treating chronic plaque psoriasis. BMJ 2000;320:963-7.
3. Chalmers RJ, O'Sullivan T, Owen CM, Griffiths CE. Interventions for guttate psoriasis. Cochrane Database Syst Rev 2000;CD001213.
4. Griffiths CE, Clark CM, Chalmers RJ, Li Wan Po A, Williams HC. A systematic review of treatments for severe psoriasis. Health Technol Assess 2000;4:1-125.
5. Owen CM, Chalmers RJ, O'Sullivan T, Griffiths CE. Antistreptococcal interventions for guttate and chronic plaque psoriasis. Cochrane Database Syst Rev 2000:CD001976.

**1999**

1. Pasker-de Jong PC, Wielink G, van der Valk PG, van der Wilt GJ. Treatment with UV-B for psoriasis and nonmelanoma skin cancer: a systematic review of the literature. Arch Dermatol 1999;135:834-40.

**1997**

1. Spuls PI, Witkamp L, Bossuyt PM, Bos JD. A systematic review of five systemic treatments for severe psoriasis. Br J Dermatol 1997;137:943-9.
