# Supplementary material for: Abstract analysis method facilitates filtering low-methodological quality and high-bias risk systematic reviews on psoriasis interventions
Source: BMC Med Res Methodol. 2017 Dec 29;17:180. doi: 10.1186/s12874-017-0460-z (PMC5747101; doi:10.1186/s12874-017-0460-z)
Supplement: Supplementary file 6 — Appendix 6. PRISMA-A IRR using Fleiss’ Kappa for two raters. (DOC 16 kb) [file 12874_2017_460_MOESM6_ESM.doc]

**Title**: Abstract analysis method facilitates filtering low-methodological quality and high-bias risk systematic reviews on psoriasis interventions

**Authors**: Francisco Gómez-García, Juan Ruano, Macarena Aguilar-Luque, Patricia Alcalde-Delgado, Jesús Gay-Mimbrera, José Luis Hernández-Romero, Juan Luis Sanz-Cabanillas, Beatriz Maestre-López, Marcelino González-Padilla, Pedro J. Carmona-Fernández, Antonio Vélez García-Nieto, and Beatriz Isla-Tejera

**Table.** PRISMA-A IRR using Fleiss' Kappa for two raters.

| Item | Fleiss' Kappa |
| --- | --- |
| PEA1 | 0.869 |
| PEA2 | 0.493 |
| PEA3 | 0.336 |
| PEA4 | 0.564 |
| PEA5 | 0.801 |
| PEA6 | 0.841 |
| PEA7 | 0.346 |
| PEA8 | 0.081 |
| PEA9 | 0.548 |
| PEA10 | 0.266 |
| PEA11 | 0.663 |
| PEA12 | 0.228 |
| Total score | 0.776 |
