# Supplementary figures and images for: Abstract analysis method facilitates filtering low-methodological quality and high-bias risk systematic reviews on psoriasis interventions
Source: BMC Med Res Methodol. 2017 Dec 29;17:180. doi: 10.1186/s12874-017-0460-z (PMC5747101; doi:10.1186/s12874-017-0460-z)

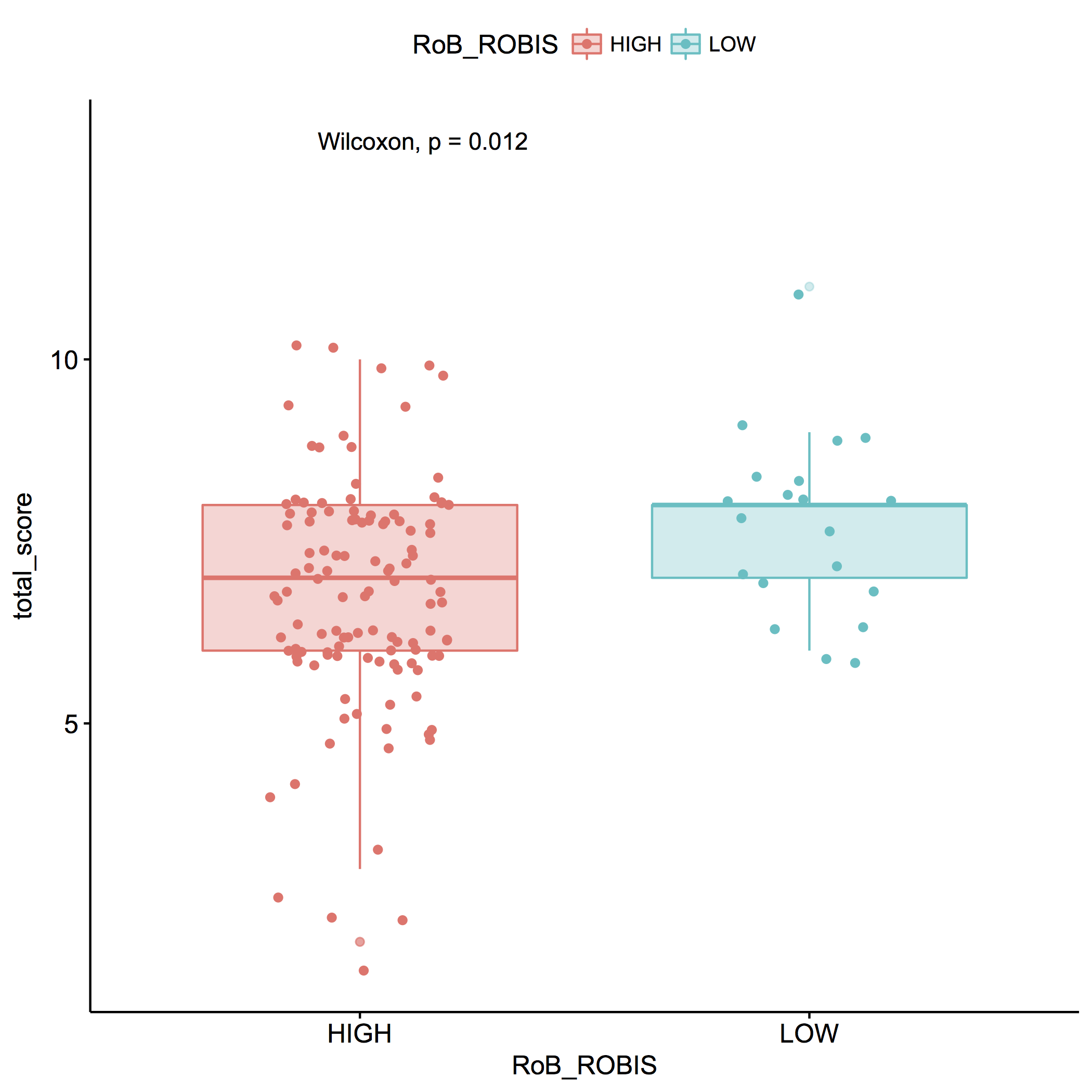

Supplement: Supplementary file 7 — Supplementary graph S1. Analysis of total PRISMA-A scores by risk of bias levels. (TIFF 17203 kb) [file 12874_2017_460_MOESM7_ESM.tiff]

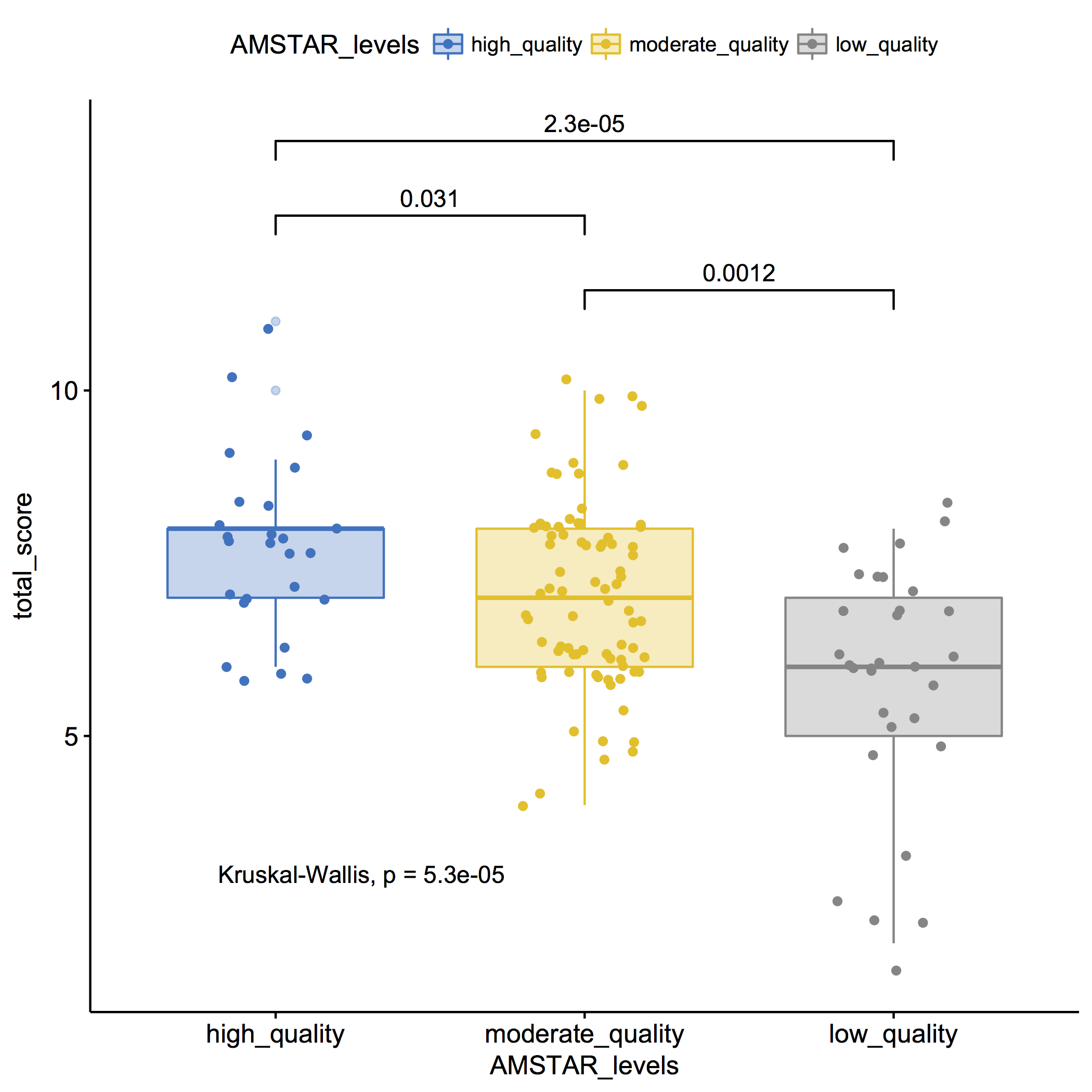

Supplement: Supplementary file 8 — Supplementary graph S2. Analysis of total PRISMA-A scores by methodological quality levels. (TIFF 17203 kb) [file 12874_2017_460_MOESM8_ESM.tiff]

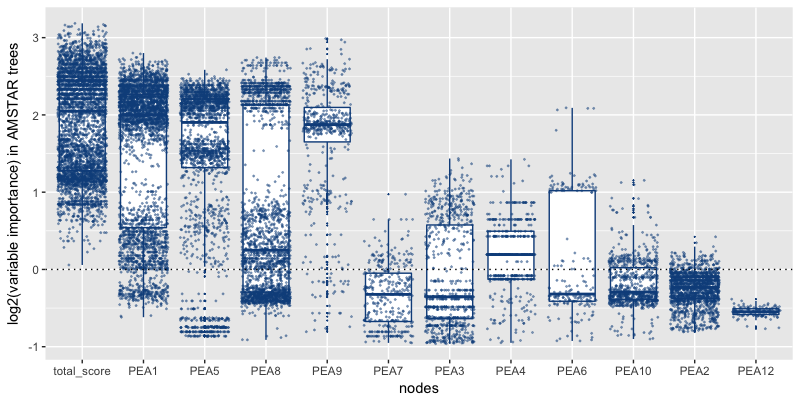

Supplement: Supplementary file 9 — Supplementary graph S3. Ranked variable importance based on mean values after running 2000 random AMSTAR-based classification tree models. (TIFF 1249 kb) [file 12874_2017_460_MOESM9_ESM.tiff]

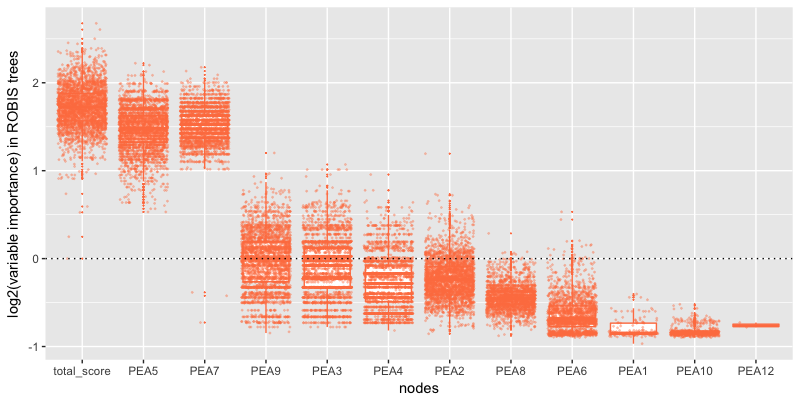

Supplement: Supplementary file 10 — Supplementary graph S4. Ranked variable importance based on mean values after running 2000 random ROBIS-based classification tree models. (TIFF 1249 kb) [file 12874_2017_460_MOESM10_ESM.tiff]
